# Haplotype block partitioning as a tool for dimensionality reduction in SNP association studies

**DOI:** 10.1186/1471-2164-9-405

**Published:** 2008-08-29

**Authors:** Cristian Pattaro, Ingo Ruczinski, Danièle M Fallin, Giovanni Parmigiani

**Affiliations:** 1 Unit of Genetic Epidemiology and Biostatistics, Institute of Genetic Medicine, European Academy, Viale Druso 1, I-39100, Bolzano, Italy; 2 Department of Biostatistics, Johns Hopkins Bloomberg School of Public Health, Baltimore, MD 21218, USA; 3 Department of Epidemiology, Johns Hopkins Bloomberg School of Public Health, Baltimore, MD 21218, USA; 4 The Sidney Kimmel Comprehensive Cancer Center, Johns Hopkins University, Baltimore, MD 21205, USA

## Abstract

**Background:**

Identification of disease-related genes in association studies is challenged by the large number of SNPs typed. To address the dilution of power caused by high dimensionality, and to generate results that are biologically interpretable, it is critical to take into consideration spatial correlation of SNPs along the genome. With the goal of identifying true genetic associations, partitioning the genome according to spatial correlation can be a powerful and meaningful way to address this dimensionality problem.

**Results:**

We developed and validated an MCMC Algorithm To Identify blocks of Linkage DisEquilibrium (MATILDE) for clustering contiguous SNPs, and a statistical testing framework to detect association using partitions as units of analysis. We compared its ability to detect true SNP associations to that of the most commonly used algorithm for block partitioning, as implemented in the Haploview and HapBlock software. Simulations were based on artificially assigning phenotypes to individuals with SNPs corresponding to region 14q11 of the HapMap database. When block partitioning is performed using MATILDE, the ability to correctly identify a disease SNP is higher, especially for small effects, than it is with the alternatives considered.

Advantages can be both in terms of true positive findings and limiting the number of false discoveries. Finer partitions provided by LD-based methods or by marker-by-marker analysis are efficient only for detecting big effects, or in presence of large sample sizes. The probabilistic approach we propose offers several additional advantages, including: a) adapting the estimation of blocks to the population, technology, and sample size of the study; b) probabilistic assessment of uncertainty about block boundaries and about whether any two SNPs are in the same block; c) user selection of the probability threshold for assigning SNPs to the same block.

**Conclusion:**

We demonstrate that, in realistic scenarios, our adaptive, study-specific block partitioning approach is as or more efficient than currently available LD-based approaches in guiding the search for disease loci.

## Background

After emerging as one of the main sources of subject-specific variation in the human genome, Single Nucleotide Polymorphisms (SNPs) are now routinely used to investigate the role of genetics in a wide spectrum of diseases [1]. The number of known SNPs is continuously growing and it is presently approaching twelve million . Technological progress is now enabling the genotyping of up to one million SNPs at a time, a number also expected to increase rapidly. This provides scientists with a considerable amount of information for the study of gene-disease associations [2]. The ability to identify associations by statistical analyses of SNP data is challenged by such high dimensionality. Strategies to organize SNP information for discovery of disease susceptibility loci have been proposed [3]. Some of these methods are especially useful when dealing with binary covariates [4], while others require exceptional computer power [5].

By studying the distribution of Linkage Disequilibrium (LD) across the genome, several authors observed that LD is related to the distance between markers [6-10]. The relationship between intermarker distance and LD does not follow a regular pattern and is related to the particular location in the human genome [11]. From these observations, it has been suggested that genetic information could be clustered into smaller sets of genomic regions [12-15] possibly separated by recombination hot spots [16]. Although the exact genetic basis for the existence of these regions is still controversial, empirically, the statistical dependence of neighboring SNPs was shown to be high. The evidence that SNPs cluster more than by chance alone suggests that treating SNPs as independent entities in association studies could be inefficient, and prone to missing true loci if multiple testing adjustments are applied. Recently, haplotype block partitioning was successfully used to accommodate the multiple testing concern while detecting genetic association in prostate cancer [17]. However, block partitioning methods differ substantially in their results [18,19]. Most comparisons between blocking methods have focused on their similarity in boundary calling or SNP membership, rather than on their ability to detect true associations.

In the present article we develop and validate a new methodology for DNA block partitioning, with a focus on improving power for association studies. Partitioning is viewed pragmatically as a genetically motivated approach to address the challenge of dimensionality. Our goal is to improve power in multiple testing and to make association testing units that are biologically meaningful. We consider each block as a single entity, by estimating a within block haplotype, thus reducing a sequence of *S *consecutive SNPs into *K *consecutive haplotype blocks. For inference on blocks we propose a probabilistic approach based on the LD map: the key idea is that pairwise LD statistics can arise from one of two separate probability distribution functions, one being the LD distribution, the other the independence distribution. This is, of course, a simplification because real LD is not binary, but this assumption has been the essence of the haplotype blocking concept. From this standpoint, blocking is similar to a classification problem and can be handled using an optimal Bayes classifier. The result is a vector of probability scores for each candidate block border SNP.

To implement this plan, we developed an MCMC Algorithm To Identify blocks of Linkage DisEquilibrium (*MATILDE*) and a framework for using *MATILDE *partitions in genetic association analysis. Our implementation presents several advantages over existing approaches, including: a) the estimation of the distribution of chance LD is specific to the population, the technology and the sample size of the study considered; b) the uncertainty about block boundaries and about whether any two SNPs are in the same block is assessed probabilistically, and c) the option for users to tune the probability threshold for assigning SNPs to the same block.

From the perspective of association studies, a block partitioning algorithm is more appealing than other ones if it provides the researcher higher chance to detect a SNP truly associated to the study trait. With detection of association in mind, we compared *MATILDE *and the most commonly used methods for haplotype block partitioning with respect to their ability to capture truly associated SNPs, rather than on boundary or membership agreement as in previous comparisons [18-20].

## Results

In our analysis we considered a representative data set from the HapMap project [21] (release 2005-09 phase II 6 chr). We considered the first 500 non redundant SNPs in region 14q11, with minor allele frequency (MAF) greater than 0.05 and Hardy Weinberg Equilibrium (HWE) at *α *= 0.01. For simplicity, we focused on unrelated individuals from a homogeneous population, by choosing the 45 Japanese, who represent the largest group of unrelated individuals within HapMap. On this data set we first carried out descriptive comparisons of block partitioning approaches, and then we performed controlled simulated experiments to assess the ability of our method to identify disease loci.

### Block partitioning of HapMap data

**Figure 1:**
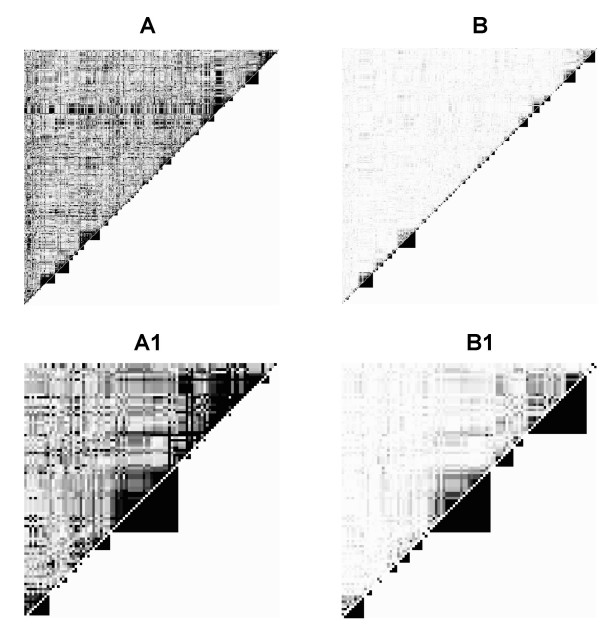
**Linkage disequilibrium on the studied region and block recognition**. Linkage Disequilibrium map of 500 SNPs spanning 2 Mb of the q11 region on Chromosome 14, based on the 45 Japanese subjects in the HapMap project [21]. We selected SNPs having a minor allele frequency of at least 5% and showing evidence of Hardy Weinberg Equilibrium. **A**. The upper triangle shows the values for |*D*'|, and the lower triangle shows the blocks estimated using *MATILDE *on the |*D*'| values. **B**. The upper triangle shows the *r*^2 ^values, and the lower triangle shows the blocks estimated using *MATILDE *on the *r*^2 ^values. **A1**. A zoom on the last 100 SNPs of panel A. **B1**. A zoom on the last 100 SNPs of panel B.

To illustrate how *MATILDE *captures LD-block information, we compared it to commonly used methods for block partitioning. Among the many methods available, we chose the limited haplotype diversity method by Patil *et al*. [13] and extended by Zhang *et al*. [22], as implemented in the HapBlock software [23] (HapBlock), and the three LD-based methods implemented in the Haploview software [24]: the Gabriel *et al*. approach [15] (DprimeCI), the Solid Spine of LD (SSD), and the four gamete test [25] (4Gamete). The computational speed of *MATILDE *was comparable to that of the HapBlock algorithm, with both being significantly slower than the rest. As expected, we observed pronounced differences in the LD map, depending on the LD statistic (Figs. 1A and 1B, upper triangles). When LD was estimated with |*D*'|, many contiguous SNPs were clustering in blocks, but strong LD was also observed between very distant SNPs, in a pattern characterized by noisy stripes. This trend is clearer when zooming in on the region from the 400^*th *^to the 500^*th *^SNP (Fig. 1A1). This made identification of block partitions more difficult. A cleaner picture was given by *r*^2 ^(Figs. 1B and 1B1), which identified a few big blocks, interspersed by a number of smaller ones, and areas with no blocks.

After 100,000 iterations of *MATILDE*, the posterior distribution of LD blocks resulted in the partition represented by the triangles on the lower right of the four panels of Fig. 1. The representation is based on a threshold of 0.5 on the marginal probability that each location is a block boundary: *MATILDE *isolated plausible LD blocks when based on *r*^2^, while the noise in |*D*'| results in a less appealing partition. The number of estimated blocks was 114 with *r*^2 ^and 215 with |*D*'|, including singletons. On the same data, HapBlock estimated 53 bigger blocks, DprimeCI 284 (217 of which were singletons). Intermediate values were observed when 4Gamete and SSD were used.

When increasing the sample size from 45 to 1000, using a resampling approach, the number of blocks estimated by DprimeCI decreases slightly from 209 to 191 (*CV *= 4.4%). 4Gamete and SSD were stable (*CV *< 2.0%), while HapBlock (*CV *= 3.0%) was intermediate. *MATILDE *with *r*^2 ^and a 0.5 probability cutoff for block boundaries had a *CV *of 3.7%. The relatively high variation of DprimeCI and *MATILDE *reflects their ability to take advantage of a more favorable signal-to-noise ratio to provide a more refined block partition.

A different trend was observed when *MATILDE *was applied to |*D*'|. With increasing sample size the number of blocks quickly degenerates to 1. This effect can be explained by a pronounced clustering of |*D*'| values to the maximum, which amplifies noise patterns at distant loci. This "ceiling effect" was also reported in a study comparing population recombination rates [26]. The ceiling effect is sensitive to noise, especially when the sample size is small or the allele frequency is extreme, in which case many observed high disequilibrium pairs would only be due to missing allelic combinations at one locus. Using *r*^2 ^results in a much reduced sensitivity to this problem [27,28]. For this reason, we only used *r*^2 ^in the simulation studies.

**Figure 2:**
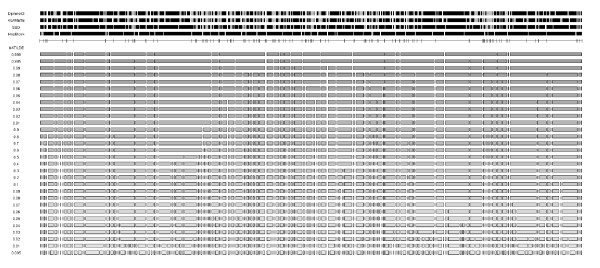
**Comparison of the block partitions on a simulated sample of 1000 subjects**. The method used is indicated on the left. On the fifth, unlabeled line, ticks are at the positions where at least three of the four methods above it agreed. *MATILDE *block structures are reported at different probability cutoffs.

An overview of the partitions obtained with each method is given in Fig. 2, for a sample size of 1000. By modulating the probability cutoff, *MATILDE *can generate a fine partition, as do LD-based methods, or a coarse one, as HapBlock (see *Additional files *1, 2, 3, *and *4 for additional sample sizes). *MATILDE *proved stable over varying cutoff, with little variation in the break points occurring for cutoffs between 0.1 and 0.9. In most instances, *MATILDE *estimated fewer single-SNP blocks than DprimeCI and 4Gamete, but a greater number of smaller blocks than HapBlock. Moderate to good agreement of break points was observed between DprimeCI, SSD, and 4Gamete: *κ *between DprimeCI and 4Gamete ranged between 0.67 and 0.76, depending on sample size; *κ*'s between SSD and DprimeCI were 0.52–0.60; while they were 0.48–0.53 between SSD and 4Gamete. DprimeCI, SSD, and 4Gamete were not in agreement with HapBlock (*κ *< 0.10 under all conditions). Generally, *MATILDE *was in an intermediate position between the LD-based approaches and HapBlock. *κ *between *MATILDE *and HapBlock was low but not null, often taking values greater than 0.10. When comparing *MATILDE *to the three LD-based approaches, we observed that *κ *was nearly the same, usually ranging between 0.20 and 0.50. The highest agreement was observed between *MATILDE *and SSD. In general, as the probability cutoff increased, the agreement between *MATILDE *and DprimeCI, 4Gamete, and SSD decreased. When HapBlock was considered, the agreement with *MATILDE *was higher for central probability cutoffs (see *Additional file *5 for an extensive overview). When a break point was concomitantly recognized by the common methods, it was typically detected by *MATILDE *as well.

### Comparison of performance in association studies

**Figure 3:**
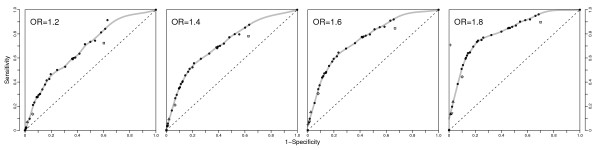
**Comparison of methods' sensitivity and specificity**. Data refer to the simulation of 500 cases and 500 controls assuming a dominant model. Each panel reports the sensitivity/specificity tradeoff for DIprimeCI (triangle), 4Gamete (reversed triangle), the SSD (diamond), HapBlock (square) and *MATILDE *(represented by points on the ROC curves, graphed as circles, and a smooth estimate of the ROC curve). In addition an allele-based single-SNP association analysis is represented by an "x" while the genotype-based single-SNP association analysis is represented by a "+". Four effect sizes were considered: the OR is 1.2, 1.4, 1.6 and 1.8, respectively.

In our simulation studies, described in detail in the Methods section, we generated artificial case-control studies with a single disease SNP, using two genotype-phenotype association models (dominant or recessive) and a range of odds ratios and sample sizes. We applied this approach in turn to all SNPs in the chosen region. This strategy preserves the observed LD in the HapMap sample. After estimating within block haplotypes, we used the likelihood ratio statistics (LRS) applied to the marginal distribution of haplotypes for each block, i.e., we performed a haplotype-based comparison rather than a diplotype-based comparison, such that each individual contributes two haplotypes, rather than one diplotype to the statistic. SNPs not in a block were considered a block of size one and in this situation, the LRS was an allelic SNP test. The sensitivity and specificity for detecting the causal SNP are reported in Fig. 3. For each method, block, and simulated dataset, we declare a positive if the p-value, after multiple testing adjustment with the Benjamini-Hochberg method [29], is smaller than .05. *MATILDE *can be used at different cutoffs for the probability that a SNP is a boundary point between blocks. Varying this threshold generates the receiver operating characteristic (ROC) curve shown. The other methods produce a single sensitivity/(1-specificity) pair. DprimeCI, 4Gamete and SSD had high specificity for all OR's, but very low sensitivity. At the other extreme, sensitivity was generally high for HapBlock, but this method had a poor specificity thus giving a high number of false positives. *MATILDE *was performing generally at equal or better sensitivity/specificity tradeoffs than the existing methods, and had the additional advantage that it could be tuned to have a higher sensitivity than the LD-based approaches. When compared to HapBlock, for p-value thresholds that achieve the same sensitivity level, *MATILDE *had about 10% greater specificity, and for the same specificity, nearly half the probability of missing a true effect – a practically important difference especially in screening studies. A better performance of *MATILDE *over other methods was observed for all sample sizes considered, as shown in the *Additional files *6, 7, 8, *and *9.

In addition to blocking approaches, we performed two types of single-SNP association analysis: allele-based, indicated with an 'x' in the graph, and genotype-based, indicated with an '+' in the graph. These are described in more detail in the *Methods *section. While the genotype-based analysis is more appropriate and more common in practice, the allele-based single-SNP analysis is reported because it is more directly comparable with the blocking methods, as it does not use phase information. Any gains seen in comparing the "x" with the blocking algorithms can be attributed to blocking. The sensitivity of the allele-based single-SNP analysis is zero in all scenarios, though some positive calls would be made at a higher false discovery rate (FDR) of 0.1. In practice, even in SNP-by-SNP studies, SNPs in close proximity with the one with the lowest p-value may be examined closely, as SNPs close to the causal SNP may have low p-values as a result of linkage disequilibrium. To capture this practice, we relaxed our definition of a "correct call" in our sensitivity/specificity calculations by considering as true positives all loci who were within a given distance from the causal SNP, and satisfied the FDR threshold. We examined SNP windows of 1, 2, 3, and 4 SNPs on each side. In all cases, results were similar to those reported in Fig. 3, and the gain in sensitivity was very modest.

**Figure 4:**
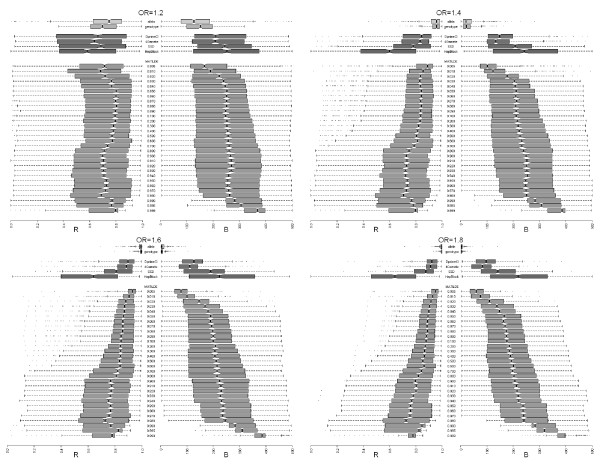
**R and B distribution**. Parallel distribution of the statistics R (relative position of the block containing the right SNP) and B (number of SNPs belonging to blocks classified not worse than the true SNP) for a sample size of 500 cases and 500 controls. Four effect sizes were considered: the OR is 1.2, 1.4, 1.6 and 1.8, respectively. For each panel, the results of simulation with the allele-based single-SNP, the genotype-based single-SNP analysis, the four common methods (DprimeCI, 4Gamete, SSD and HapBlock) and the *MATILDE *at various cutoff thresholds are listed.

Fig. 4 summarizes results obtained using two additional comparison criteria that better highlight important properties of the blocking approaches. Criterion R represents the ratio of the rank of the block including the causal SNP, and the total number of blocks. On the left sides of the four panels, we reported the distribution of *R *at ORs ranging from 1.2 to 1.8. The better methods are those with distributions of *R *closer to 1. Boxplots represent variability over simulated datasets. For small effects, that is *OR *= 1.2, the median *R*'s for DprimeCI, SSD and 4Gamete were comparable, and all are higher than for HapBlock. The median for *MATILDE *at several cutoffs was the highest, by a sizeable margin, even when compared to the single SNP analysis. This is because, for small effects, there are often several SNPs that are ranked better than the causal one in the single locus analysis. At increasing OR's the performance of DprimeCI and 4Gamete improved and for values bigger than 1.4, they were on average slightly better than *MATILDE*. For effects ≥ 1.4, the analysis at single locus outperformed the other methods (see *Additional files *10, 11, 12, *and *13 for additional sample sizes).

Criterion *B *is the count of SNPs belonging to blocks ranked as high or higher than the block including the correct SNP (Fig. 4, right sides). Lower values of B are preferable. For small OR's, the blocking methods performed comparably, with the exception of *MATILDE *at cutoffs ≤ 0.1, which had a better performance. At higher OR's (see Additional files 10, 11, 12, and 13) the methods with the highest number of single-SNP blocks (DprimeCI, 4Gamete and *MATILDE *with cutoffs ≤ 0.1) had a significantly better performance than SSD, HapBlock and *MATILDE *with bigger cutoffs. As expected, the single SNP analysis performed better than blocking methods by this criterion. Consistently, low-cutoff *MATILDE *provided the best performance in both *R *and *B*.

## Discussion

Overall, our experimental results suggest that probabilistic modeling of LD patterns is a useful approach to summarize a high dimensional collection of SNPs into a smaller set of haplotype blocks when searching for disease-related loci. Our methodology, implemented in the *MATILDE *program, adapts to the available data, provides an assessment of uncertainty, and can be used flexibly as a dimension reduction tool compared to the alternatives available so far. In our HapMap-based simulation experiments, *MATILDE *showed the best ability to rank loci when looking for small effect sizes. This is a critical strength, since most SNP association studies involve small effect sizes. An important, empirical example, in this sense, was recently illustrated in the field of prostate cancer [30]. DprimeCI and 4Gamete perform well in ranking, though at the cost of a large number of single-SNP blocks, which makes these methods less efficient when using multiple comparisons corrections. *MATILDE *also provides significant gains in sensitivity when a low specificity is appropriate – as in SNP screening studies – and is comparable to the other methods considered in the high specificity range.

For a broad range of sample sizes and effect sizes, traditional single-SNP analyses fail to find the causal locus. These analyses only become effective when the sample size is greater than 1000 and the effect size is at least 1.8 – a rare case in genomics. Otherwise, grouping SNPs into blocks with any method is a better solution. This conclusion reinforces the suggestion of Zhang *et al*. [31] that haplotype-based analysis can be much more powerful than single locus analysis. Their study was based on HapBlock. In our simulations *MATILDE *shows better performance than HapBlock, so the case for blocking is now stronger.

While our simulations consider a large number of scenarios (over 850,000) and are closely mimicking real data, there remain some limitations. First, because of the computational burden, it would have been prohibitive to consider the joint distribution at two chromosomes after blocking. Thus our comparisons are based on the simpler unphased haplotype estimation, whereby each subject contributes two separate haplotypes, and association is assessed by comparing the distribution of cases' haplotypes to that of the controls. This approach is still the most prevalent in applications, but may negatively affect the performance of all blocking methods, and may favor the single marker analysis for big effects, especially with regard to the *R *and *B *performance criteria. To explore the potential gains in efficiency that can be expected when using the phase information, we carried out a genotype-based single-SNP analysis. This is indicated by a '+' in Fig. 3 and should be compared only to the 'x' symbol, which represents the results of the allele-based single-SNP analysis. We also reported both analyses in Fig. 4. For *R *and *B *the results are similar, while a difference is observed at an OR of 1.8 in Fig. 3. The latter, however is partly the result of a sensitivity to the choice of the significance level, and is not as pronounced when a stricter level of .01 is required.

As a second limitation, we focused our comparison on the most commonly used block partitioning methods. Minimum Description Length (MDL) methods [32-34], including the *MDBlock *implementation [32], have also been shown to reliably locate boundaries between blocks at regions of rapid LD decay, and produce block partitions of intermediate size between those of LD-based approaches and those given by limited haplotype diversity methods. Additionally, future work could consider the comparison between blocking and using tagging SNPs. Two useful approaches, *HaploBlockFinder *[35] and *htSNPer *[36], produce both haplotype or LD blocks, and tagging SNPs. As they yield block partitions similar to those of methods already covered by Haploview and HapBlock, they were not considered here, but would be natural choices if tagging SNPs were studied. Lastly, the iHAP (integrated haplotype analysis pipeline) [37] integrates several block partitioning and tagging SNPs methods with web resources for gene finding. It was explicitly defined to mine the HapMap dataset by means of the HapBlock software and it has not the aim to process user's genotype data.

Our results include a descriptive analysis of the agreement among blocking approaches. Our goal is to provide further intuition about the reasons behind the performance of different blocking methods in identifying disease SNPs, rather than fully characterizing their behavior from a population genetics viewpoint. In our study, block partitioning is an intermediate step towards identifying genotype-phenotype associations, which is ultimately assessed through statistical models. This bypasses the need for a gold standard for haplotype blocks, and also brings the evaluation closer to practical study goals. To account for the potential instability of estimated blocks when small sample sizes are taken [20], we also examined large sample sizes.

While several measurements of agreement between blocks are available in the literature, we chose the simple *κ *statistic on the between block break points. Alternatively, the *SB*_2 _statistic [38] would have been useful when comparing two populations one of which is considered less diverse than the other one, that is, in the case where block boundaries could vary among populations. In our case, however, we were considering a homogeneous sample of subjects from the same geographical location and testing different methods over the same small chromosomal segment. Our results were consistent with those of Schwartz *et al*. [18] who defined an agreement statistic based on the number of shared boundaries. The block partition given by *MATILDE *was more similar to the LD-based methods than to HapBlock. Since the *MATILDE *block estimation is based on the LD map, this finding was not surprising. Other authors [19] compared the LD-based method of Gabriel *et al*. [15] and the limited haplotype diversity method of *Patil et al*. [13], in the Zhang *et al*. formulation [22]: they found that block partitions given by the two methods were different, strongly dependent on minor allele frequencies, and sensitive to changes in the algorithms' parameters. We confirm the previous observation [18,19] that the method from Gabriel *et al*. [15] generates a higher number of smaller blocks than that of Patil *et al*. [13].

Block partitioning criteria can potentially perform at different levels of sensitivity and specificity in different populations. Spatial correlation in the genome can be influenced by a variety of factors, including demographic history and recombination hotspots [39]. Depending on how these factors contribute to the block structure in a population, different partitioning criteria may differ in their ability to identify associations. An assessment of how different methods could perform in populations with different demographic evolution is an interesting question for further research. *MATILDE*, however, differs from biologically based methods such as the four gamete rule [25] as it was designed pragmatically, without any reference to biological theories about the origin of blocks. We can speculate that *MATILDE *may be more powerful than methods based on biological hypotheses in situations where there is noise in the LD pattern, as is the case of outbred populations. In isolated populations, where population growth followed a bottleneck event, haplotype heterogeneity is much smaller and individuals share longer chromosomal regions. When this situation is also accompanied by a reduced number of external individuals, one may expect less noise in the LD pattern, and most of the block partitioning methods should give more similar results.

Our method allows users to specify a pairwise measure of LD. This choice matters: in our analysis *MATILDE*'s performances varied depending on whether *r*^2 ^or |*D*'| was used. Both measures have a clear genetic interpretation. The expected value of *r*^2 ^is a direct function of the population recombination rate, and *r*^2 ^is the standard *χ*^2 ^test statistic divided by the number of chromosomes. Thus, it is a natural candidate for testing the disequilibrium between loci [9,40,41]. Strengths and limitations of |*D*'| have already been described [28]. LD can be assessed by many other statistics. An extensive list is provided by Devlin and Risch [42]. An example is Levin's population attributable risk [43]. Statistics that show a robust behavior in case control studies are the difference in proportion *d *suggested by Nei and Li [44], the odds ratio, and the Yule's *Q *[45]; *d *and *Q *are bounded between 0 and 1 and between -1 and 1, respectively. More recently, entropy was suggested as a measure of LD for multiallelic loci [46], and the volume measures of LD proved to be robust in case of small samples [47]. In addition, potential candidates are the Morton's rho [48], which models LD by a linear mixture of SNPs under non-LD and in perfect LD, and the Delta statistic [49] which is less noisy than *r*^2 ^and *D*', and is robust to allele frequency.

The ability to adapt to SNP density is an advantage of using a Bayes classifier like *MATILDE*. Marker density affects the LD distribution [50], though this is not an issue when clustering is used only as a dimension reduction step. In our formulation, block partitioning is related to the specific set of SNPs typed. This is different from estimating blocks on the basis of recombination hotspots [51], which aims at uncovering an underlying genetic structure.

While our implementation was successful as a proof of principle, additional work remains necessary before the full potential of dimension-reduction by blocking can be realized. For example, computational obstacles remain before the current implementation of *MATILDE *can be used efficiently on studies of the size of current genome-wide association analyses. We plan to address these computational issues in future versions of the program.

Finally, we hope that the idea of using probabilistic blocking for dimension reduction of DNA information can in the future become the foundation for a comprehensive analysis, including haplotype reconstruction, missing data imputation, and modeling of the genotype-phenotype relationship. It has been shown that the best method for haplotype reconstruction when the phase is unknown is also probabilistic and based on MCMC [52,53]. The issue of integrating block partitioning and haplotype reconstruction was already undertaken by some authors [54,55]. Additionally, a potentially important extension available within an integrated approach is the ability to construct blocks that optimally capture association signal, a feature which is not presently implemented in our approach.

## Conclusion

We demonstrated that at low signal-to-noise ratio, blocking SNP's via a classification approach can lead to significant increases in efficiency in identifying disease related loci. For this task, we provided a flexible methodology and software.

## Methods

### A probabilistic formulation of LD maps

LD is the non-random association between alleles at different loci [56]. Let us now consider a sequence of *S *SNPs, ordered by chromosomal location. The set of all the *S*(*S *- 1)/2 pairwise LD statistics is Θ = {*θ*_*ij*_, *i *= 1, ..., *S *- 1; *j *= 2, ..., *S*}. Note that *θ *can be any measure of LD among those varying in [0, 1] [42].

Denote by Θ_1 _the subset of *θ*s estimated from SNPs in true LD, and by Θ_0 _the subset of *θ*s estimated from SNPs which are not in LD. Since no other intermediate option is allowed between the LD and the absence of LD status, then Θ_1 _∪ Θ_0 _≡ Θ. Under the assumption that two SNPs are in LD only if they belong to the same haplotype block, the partition of Θ can be uniquely identified by a binary vector *γ* = [*γ*_1_, *γ*_2_, ..., *γ*_*i*-1_, *γ*_*i*_, *γ*_*i*+1_, ..., *γ*_*S*_, *γ*_*S*+1_]*' *where *γ*_*i *_= 1 means that the a border of a haplotype block falls between SNPs (*i *- 1) and *i*; *γ*_*i *_= 0 means that SNP (*i *- 1) and SNP *i *belong to the same block. By definition, *γ*_1 _= 1 and *γ*_*S*+1 _= 1. SNPs not belonging to any block are classified as blocks by themselves, with borders *γ*_*i*-1 _= *γ*_*i *_= 1. In the following, *γ* will be referred to as *block border vector*.

Empirical evidence and theoretical studies [57-59] showed that the distribution of the *θ*s, *f*(*θ*), is generally skewed to the right, often with a mass close to 1. The magnitude of this mass depends on the LD statistic used and on the study sample size. This property of *θ*, made us to assume that *f*(*θ*) is composed of two underlying distributions, so that

$$p(\theta_{ij}|f_{0},f_{1},\underline{\gamma })=\left\{\begin{array}{cc} f_{0}(\theta_{ij}) & \text{if}\theta_{ij}\in \Theta_{0}(\underline{\gamma })\\ f_{1}(\theta_{ij}) & \text{if}\theta_{ij}\in \Theta_{1}(\underline{\gamma }) \end{array}\right\}$$

the *θ*s being drawn from *f*_1 _when the SNPs are in LD, from *f*_0 _otherwise.

For given *f*_0_, *f*_1_, and *γ*, and assuming conditional independence of the *θ*s, the likelihood is

(1) $$\begin{array}{lll} L_{\theta_{ij}}(\underline{\gamma }|f_{0},f_{1}) & = & p(\theta_{ij}|f_{0},f_{1},\underline{\gamma })\\ & = & \prod_{\theta_{ij}\in \Theta }p(\theta_{ij}|f_{0},f_{1},\underline{\gamma })\\ & = & \prod_{\theta_{ij}\in \Theta_{0}}p(\theta_{ij}|f_{0},\underline{\gamma })\prod_{\theta_{ij}\in \Theta_{1}}p(\theta_{ij}|f_{1},\underline{\gamma }) \end{array}$$

The assumption of conditional independence does not correspond closely to how the data are generated, and is made pragmatically, to simplify an otherwise nearly intractable problem. We consider it unlikely that this assumption will significantly affect the accuracy of the classification, although it may affect the uncertainty assessment. Alternatively, one can model the joint distribution of haplotypes directly and address blocking, for example, as a model selection problem [60]. This approach is more realistic but not yet scalable to the number of SNPs generated by current technology.

Because of the one-to-one correspondence between *γ* and {Θ_0_, Θ_1_}, the (1) can be written as

(2) $$L_{\theta_{ij}}(\underline{\gamma }|f_{0},f_{1})=\prod_{\theta_{ij}\in \Theta_{0}}f_{0}(\theta_{ij})\prod_{\theta_{ij}\in \Theta_{1}}f_{1}(\theta_{ij}).$$

*f*_0 _can be estimated non-parametrically by randomly permuting the genotypes between subjects. LD is estimated from the genotype distribution via an EM algorithm [61], and the empirical ${\hat{f}}_{0}$ is finally estimated with a kernel smoothing method [62]. As permutation affects LD estimation, these steps were repeated several times and the final estimate ${\hat{f}}_{0}$ of the density was the average of each of the densities, evaluated on a grid of 1000 percentile points.

Let's assume *θ *∈ Θ_1 _follows a Beta distribution, *θ*_*ij *_| Θ_1_, *α*, *β *~ *Beta*(*α*, *β*), such that

(3) $$f_{1}(\theta_{ij}|\alpha ,\beta )=\frac{\Gamma (\alpha ,\beta )}{\Gamma (\alpha )\Gamma (\beta )}{\left(\theta_{ij}\right)}^{\alpha -1}{\left(1-\theta_{ij}\right)}^{\beta -1}$$

with *α *> 0, *β *> 0, and assume that *β *> *α *to ensure that the mode of this distribution is greater than 0.5. Substitutions and simple algebra allows to write the log-likelihood *l*_*θ *_(*γ*, *α*, *β *|${\hat{f}}_{0}$, *f*_1_) as

(4) $$\sum_{\theta_{ij}\in \Theta_{0}}log{\hat{f}}_{0}(\theta_{ij})+\#\{\Theta_{1}\}log\frac{\Gamma (\alpha ,\beta )}{\Gamma (\alpha )\Gamma (\beta )}+(\alpha -1)\sum_{\theta_{ij}\in \Theta_{1}}log\theta_{ij}+(\beta -1)\sum_{\theta_{ij}\in \Theta_{1}}log(1-\theta_{ij})$$

with the unknown parameters being *α*, *β *and *γ*.

The (4) was explored by means of a Metropolis-Hastings algorithm, using uniform priors on all unknowns, within the constraints described above (see the Appendix for a detailed description of the algorithm). With respect to the block border vector, starting values can be chosen using a threshold criteria [10] in order to have a block border where *θ*_*i*, *i*+1 _- *θ*_*i*+1, *i*+2 _> *τ*, with *τ *that can be defined by the user on the basis of the particular LD statistic being used.

The algorithm was tested using the absolute value of the Lewontin's D-prime, |*D*'|, and the square of the correlation coefficient for 2 × 2 tables, *r*^2 ^[56,63,42]. Indeed, the described approach applies to any measure of LD between two loci.

Several tests demonstrated that, when a sufficient number of iteration is performed, starting values do not influence the results. The posterior distribution for (*γ*, *α*, *β*) was estimated after eliminating the first half of the Markov Chain, as burn-in. The chain can be used to estimate the vector of the *S *+ 1 probabilities of each point being a block border. Partitions at varying probability cutoff can be derived from these estimates.

### Software and blocking algorithm definitions

*MATILDE *was written in the R language and requires the package *genetics *[64]. The software is available at . Haploview 3.2 [24] was used for: (i) LD map estimation, (ii) genotype data cleaning, (iii) block partitioning, and (iv) for estimating the within block haplotype distributions. The blocking methods implemented in Haploview that were used in our analysis were the following: DprimeCI is the method proposed by Gabriel *et al*. [15] and based on the *D' *statistic; SSD is the Solid Spine of LD method (for a detailed description see the support documentation of the software at ); 4Gamete is the "Four Gamete Rule" by Wang *et al*. [25], which assumes that a recombination took place when all the four possible two-marker haplotypes between couples of contiguous SNPs occur. HapBlock v3.0 [23] was used for the limited haplotype diversity approach suggested by Patil *et al*. (Hapblock) [13]. While the original method was based on a greedy algorithm which did not ensure an optimal solution to the problem of block partitioning, the program is based on the dynamic programming algorithm for haplotype partitioning introduced by Zhang and colleagues [22] which guarantees to find an unique optimum. Hapblock provides the possibility to use one of three definitions of haplotype blocks: we selected the "common haplotype" option. Under this definition, "a set of consecutive SNPs with size one or more forms a block if the number of common haplotypes account for at least *a *percent of all the observed haplotypes (see the manual available at the software's homepage  for more details). For the parameters *α *and *β *we used the recommended values of 5% and 80%, respectively. R 2.6.0 [65] was used to perform the whole analysis and to interface Haploview and Hapblock.

### Descriptive analysis of block partitioning approaches

To facilitate the comparison between different methods, we defined all the single SNPs outside blocks to be blocks by themselves. This is required because with DprimeCI, 4Gamete, and SSD the SNP blocking may not be exhaustive of all the SNPs in the series. To explore the sensitivity to sample size we obtained samples of 200, 400, 600, 800, and 1000 then by drawing, with replacement, the 45 original subjects, leaving their SNP profiles unchanged, to preserve the LD structure. Empirical block structures of the study chromosomal region were obtained from all methods for each sample size. A much finer inspection was run for *MATILDE *to assess the performance under different cutoff levels (0.01, 0.02, ..., 0.1, ..., 0.9, 0.95, ...). The variability of the number of estimated blocks was assessed via the *coefficient of variation *(*CV*). The agreement between methods was assessed through the *κ *statistics [66] on the number of shared break points.

### Comparison of performance in association studies

In our simulations, we generated case-control studies each including a single disease SNP. For each SNP in the sequence, we created several artificial case-control studies each with a 1:1 ratio of cases to controls. For both dominant and recessive genotype-phenotype association models, subjects were classified into risk allele carriers and non carriers; then subjects were assigned to cases or to controls in a random way, satisfying the constraint of a pre-specified Odds Ratio (OR), that is the proportion of risk allele carriers in cases and controls was fixed in advance. ORs used are 1.2, 1.4, 1.6, 1.8, and 2.0. This was repeated for five choices of sample sizes. In this way, we covered a wide spectrum of scenarios, while preserving the empirically observed LD.

Block partition and haplotype distribution were estimated on the pooled samples. In this way we could reuse the partitions estimated in the previous section. Within block haplotype distributions were estimated using the EM algorithm [61], separately for cases and controls. Subject's chromosomes were considered to be independent so that each subject carried two haplotypes. Because blocks were determined without consideration for case status, they are not optimized statistically to maximize the block associations.

Our analysis proceeds as follows: given a haplotype block, we estimated the haplotype. Each subject contributes two phased haplotypes, one for each chromosome. Using the Likelihood Ratio Statistics (LRS) we compared the distribution of haplotypes in cases and in controls. Specifically, within the *k*^*th *^block, the LRS $G_{k}^{2}=2\sum_{i=1}^{2}\sum_{j=1}^{m_{k}}n_{ijk}log\left(\frac{n_{ijk}}{\nu_{ijk}}\right)$ was used to test the hypothesis of independence of the haplotype distribution in cases (*i *= 1) and in controls (*i *= 2), with *m*_*k *_being the number of observed haplotypes in the *k*^*th *^block, *n*_*ijk *_the observed frequency of the haplotype *j *in the group *i*, *ν*_*ijk *_the expected frequency of the haplotype *j *in group *i *under independence. For large sample sizes, $G_{k}^{2}\sim \chi_{m_{k}-1}^{2}$[67]. Because the choice of the best method, on the basis of genotype-phenotype association, depends on the study goals, the efficiency of the block partition algorithms was ranked under different criteria. First, for each block partitioning method, *G*^2 ^and the relative p-value were estimated; then, the p-values were sorted in descending order: *p* = {*p*_(1)_, ..., *p*_(*k*)_, ..., *p*_(*K*-1)_, *p*_(*K*)_}, with *K *being the number of blocks. In the following we define k* as the index of the block containing the SNP that is truly associated with the disease. For single-SNP analyses, we examined two strategies. The first one is to consider each locus as a block of length one, and apply the procedure above. For example if we have 3 subjects with genotypes, 'AA', 'Aa' and 'Aa', respectively, then the marginal allelic distribution is 'A' with frequency 4 and 'a' with frequency 2. This type of distribution will be compared across cases and controls using the LRS. We refer to this as allele-based single-SNP analysis. The second one is a genotype-based single-SNP analysis, where the marginal allelic distribution is replaced by the genotype distribution, that is: AA with frequency 1, Aa with frequency 2. The reason for considering the allele-based analysis is to allow a fair comparison with other blocking approaches, where a genotype-based analysis would have been too onerous to implement. Sensitivity/specificity comparisons are based on mimicking the association testing situation. For each method, block, and simulated dataset, we declare a positive if the p-value, after multiple testing adjustment with the Benjamini-Hochberg method [29], is smaller than .05. In more detail, let *T*_*k*, *j *_be an indicator variable for the *k*^*th *^block at the *j*^*th *^simulation: *T*_*k*, *j *_= 1 when the null hypothesis is rejected, 0 otherwise. Let *J *be the number of simulations and $k_{j}^{*}$ the indicator for the right block at the *j*^*th *^simulation, then $Se=\sum_{j=1}^{J}T_{k_{j}^{*},j}/J$ is the *Sensitivity*, that is the probability of deciding that the block k* contains the right SNP, when this is true. The *Specificity *is the probability of deciding that a block does not contain the right SNP when it actually does not contain the SNP. Thus $Sp=\frac{1}{J}\sum_{j=1}^{J}Sp_{j}$, where $Sp_{j}=\frac{1}{K_{j}-1}\sum_{k=1,k\neq k_{j}^{*}}^{K_{j}}\left(1-T_{k,j}\right)$ is the specificity at the *j*^*th *^simulation. To assess the behavior of *MATILDE *at different probability cutoffs (that is the probability to classify a specific location as a block border), a Receiver Operating Characteristic (ROC) curve fitted by means of a local polynomial regression (*loess*), each point of the curve being the sensitivity/(1-specificity) combination for one specific probability cutoff. At this scope we used the function *loess.smooth *implemented in the R package *stats*.

#### RELATIVE POSITION OF THE CORRECT BLOCK

From the standpoint of evaluating the quality of the dimension reduction methodology, it is useful to reward approaches that give a high ratio *R *= (k* - 1)/*K*, with *R *∈ [0, (*K *- 1)/*K*]. This statistic is a way to reward the method which is faster in finding the area where the right SNP is, irrespective of the dimension of blocks.

#### RELATIVE POSITION OF THE CORRECT SNP

When the dimension of blocks matters, it could be more interesting to count the number of SNPs classified as good as, or better than, the right SNP, that is $B=\sum_{k=k^{*}}^{K}\#B_{k}$, where #*B*_*k *_is the number of SNPs in the *k*^*th *^block. *B *is the number of SNPs that should be screened before discovering the true SNP, thus the smaller the *B *the better the method.

## Authors' contributions

The scientific motivation and statistical models were defined by CP and GP with the support of IR for the computational expertise and MDF for epidemiological expertise. Simulations were performed by CP. The article was written mainly by CP with substantial contribution by all the other authors. The project was supervised by GP.

## Appendix

Description of the *MATILDE*'s core algorithm.

### The Metropolis-Hastings algorithm

Here the *t*^*th *^iteration of the Metropolis-Hastings algorithm used to explore the (4) is described. The parameters (*γ*_*t*-1_, *α*_*t*-1_, *β*_*t*-1_) were updated in three steps as follows:

1^*st *^step 

i) sample *γ*_*t *_as described below;

given *γ*_*t*_, split Θ into $\Theta_{0}^{*}$ and $\Theta_{1}^{*}$

compute *l*_*t*_(*γ*_*t*_, *α*_*t*-1_, *β*_*t*-1 _| ${\hat{f}}_{0}$, *f*_1_); *r *= exp {*l*_*t *_- *l*_*t*-1_};

ii) sample *u *~ *U*(0, 1);

if *u *<*min*(*r*, 1) then {Θ_0_, Θ_1_} ← {$A_{0}^{*}$, $A_{1}^{*}$};

else *γ*_*t *_← *γ*_*t*-1 _and *l*_*t*_← *l*_*t*-1_;

2^*nd *^step

i) sample *β*_*t *_~ *U*(*β*_*t*-1 _- 1, *β*_*t*-1 _+ 1);

compute l*(*γ*_*t*_, *α*_*t*-1_, *β*_*t *_| *γ*_*t*-1_, *f*_1_); *r *= exp {l* - *l*_*t*_};

ii) sample *u *~ *U*(0, 1);

if *u *<*min*(*r*, 1) then *l*_*t*_← l*;

else *β*_*t *_← *β*_*t*-1_;

3^*rd *^step

i) sample a* ~ *U*(*α*_*t*-1 _- 1, *α*_*t*-1 _+ 1);

*α*_*t *_← *max*(*β*_*t*_, α*);

compute l*(*γ*_*t*_, *α*_*t*_, *β*_*t *_| *γ*_*t*-1_, *f*_1_); *r *= exp {l* - *l*_*t*_};

ii) sample *u *~ *U*(0, 1);

if *u *<*min*(*r*, 1) then *l*_*t *_← l*;

else *α*_*t *_← *α*_*t*-1_.

### Sampling the block border vector

At each iteration, *t*, the key point is the proposal of the new block border vector, which is sampled as follows: first, let's decide either to move a boundary (i) or to change the number of blocks. Option (i) corresponds to changing the size of two neighboring blocks, option (ii) corresponds to joining or splitting two neighboring blocks. The choice is done by sampling from a Bernoulli(*p*), with *p *defined by the user on the basis of sample size and number of SNPs.

Under the option (i), one of the existing boundaries, *γ*_*t*,1_··*γ*_*t*, *S*+1_, is sampled with equal probability; then the border is moved one step to the left or to the right at random: of the two blocks sharing the boundary, one will increase its size of one SNP, while the other will be shortened by one. When this move is chosen, the total number of blocks does not change.

Under the option (ii), one of two actions is sampled with equal probability: I) split one block: one block is sampled at random and one point inside the block is also chosen at random and turned into a border, generating two contiguous and smaller blocks; II) modify a random value of *γ*_*t*_: one point, *γ*_*t*, *i*_, between *γ*_*t*,2 _and *γ*_*t*, *S*_, is randomly chosen; if *γ*_*t*, *i *_= 0 then *γ*_*t*, *i *_← 1 (this means to join two contiguous blocks into a bigger one), else *γ*_*t*, *i *_← 0 (this is equivalent to splitting one block into two smaller ones).

## Supplementary Material

Additional file 1 Comparison of the block partitions on a simulated sample of 200 subjects. The method used is indicated on the left. On the fifth, unlabeled line, ticks are at the positions where at least three of the four methods above it agreed. *MATILDE *block structures are reported at different probability cutoffs. Click here for file

Additional file 2 Comparison of the block partitions on a simulated sample of 400 subjects. The method used is indicated on the left. On the fifth, unlabeled line, ticks are at the positions where at least three of the four methods above it agreed. *MATILDE *block structures are reported at different probability cutoffs. Click here for file

Additional file 3 Comparison of the block partitions on a simulated sample of 600 subjects. The method used is indicated on the left. On the fifth, unlabeled line, ticks are at the positions where at least three of the four methods above it agreed. *MATILDE *block structures are reported at different probability cutoffs. Click here for file

Additional file 4 Comparison of the block partitions on a simulated sample of 800 subjects. The method used is indicated on the left. On the fifth, unlabeled line, ticks are at the positions where at least three of the four methods above it agreed. *MATILDE *block structures are reported at different probability cutoffs. Click here for file

Additional file 5 Agreement between all block partitioning methods. Upper panels: pairwise *κ *s between the four most common methods, by sample size. Lower panel: *κ *s between *MATILDE *and the four most common methods, by probability cutoff (x-axis) and sample size. Symbols: triangle = DprimeCI, diamond = SSD, reverse triangle = 4Gamete, and square = HapBlock. Click here for file

Additional file 6 Comparison of methods' sensitivity and specificity. Data refer to the simulation of 100 cases and 100 controls assuming a dominant model. Each panel reports the sensitivity/specificity tradeoff for DIprimeCI (triangle), 4Gamete (reversed triangle), the SSD (diamond), HapBlock (square) and *MATILDE *(represented by points on the ROC curves, graphed as circles, and a smooth estimate of the ROC curve). In addition an allele-based single-SNP association analysis is represented by an "x" while a genotype-based single-SNP association analysis is represented by a "+". Four effect sizes were considered: the OR is 1.2, 1.4, 1.6 and 1.8, respectively. Click here for file

Additional file 7 Comparison of methods' sensitivity and specificity. Data refer to the simulation of 200 cases and 200 controls assuming a dominant model. Each panel reports the sensitivity/specificity tradeoff for DIprimeCI (triangle), 4Gamete (reversed triangle), the SSD (diamond), HapBlock (square) and *MATILDE *(represented by points on the ROC curves, graphed as circles, and a smooth estimate of the ROC curve). In addition an allele-based single-SNP association analysis is represented by an "x" while a genotype-based single-SNP association analysis is represented by a "+". Four effect sizes were considered: the OR is 1.2, 1.4, 1.6 and 1.8, respectively. Click here for file

Additional file 8 Comparison of methods' sensitivity and specificity. Data refer to the simulation of 300 cases and 300 controls assuming a dominant model. Each panel reports the sensitivity/specificity tradeoff for DIprimeCI (triangle), 4Gamete (reversed triangle), the SSD (diamond), HapBlock (square) and *MATILDE *(represented by points on the ROC curves, graphed as circles, and a smooth estimate of the ROC curve). In addition an allele-based single-SNP association analysis is represented by an "x" while a genotype-based single-SNP association analysis is represented by a "+". Four effect sizes were considered: the OR is 1.2, 1.4, 1.6 and 1.8, respectively. Click here for file

Additional file 9 Comparison of methods' sensitivity and specificity. Data refer to the simulation of 400 cases and 400 controls assuming a dominant model. Each panel reports the sensitivity/specificity tradeoff for DIprimeCI (triangle), 4Gamete (reversed triangle), the SSD (diamond), HapBlock (square) and *MATILDE *(represented by points on the ROC curves, graphed as circles, and a smooth estimate of the ROC curve). In addition an allele-based single-SNP association analysis is represented by an "x" while a genotype-based single-SNP association analysis is represented by a "+". Four effect sizes were considered: the OR is 1.2, 1.4, 1.6 and 1.8, respectively. Click here for file

Additional file 10 Parallel distribution of the statistics R (relative position of the block containing the right SNP) and B (number of SNPs belonging to blocks classified not worse than the true SNP) for a sample size of 100 cases and 100 controls. Four effect sizes were considered: the OR is 1.2, 1.4, 1.6 and 1.8, respectively. For each panel, the results of simulation with the allele-based single-SNP method, the genotype-based single-SNP analysis, the four common methods (DprimeCI, 4Gamete, SSD and HapBlock) and the *MATILDE *at various cutoff thresholds are listed. Click here for file

Additional file 11 Parallel distribution of the statistics R (relative position of the block containing the right SNP) and B (number of SNPs belonging to blocks classified not worse than the true SNP) for a sample size of 200 cases and 200 controls. Four effect sizes were considered: the OR is 1.2, 1.4, 1.6 and 1.8, respectively. For each panel, the results of simulation with the allele-based single-SNP method, the genotype-based single-SNP analysis, the four common methods (DprimeCI, 4Gamete, SSD and HapBlock) and the *MATILDE *at various cutoff thresholds are listed. Click here for file

Additional file 12 Parallel distribution of the statistics R (relative position of the block containing the right SNP) and B (number of SNPs belonging to blocks classified not worse than the true SNP) for a sample size of 300 cases and 300 controls. Four effect sizes were considered: the OR is 1.2, 1.4, 1.6 and 1.8, respectively. For each panel, the results of simulation with the allele-based single-SNP method, the genotype-based single-SNP analysis, the four common methods (DprimeCI, 4Gamete, SSD and HapBlock) and the *MATILDE *at various cutoff thresholds are listed. Click here for file

Additional file 13 Parallel distribution of the statistics R (relative position of the block containing the right SNP) and B (number of SNPs belonging to blocks classified not worse than the true SNP) for a sample size of 400 cases and 400 controls. Four effect sizes were considered: the OR is 1.2, 1.4, 1.6 and 1.8, respectively. For each panel, the results of simulation with the allele-based single-SNP method, the genotype-based single-SNP analysis, the four common methods (DprimeCI, 4Gamete, SSD and HapBlock) and the *MATILDE *at various cutoff thresholds are listed. Click here for file
