# Supplementary figures and images for: Haplotype block partitioning as a tool for dimensionality reduction in SNP association studies
Source: BMC Genomics. 2008 Aug 29;9:405. doi: 10.1186/1471-2164-9-405 (PMC2547855; doi:10.1186/1471-2164-9-405)

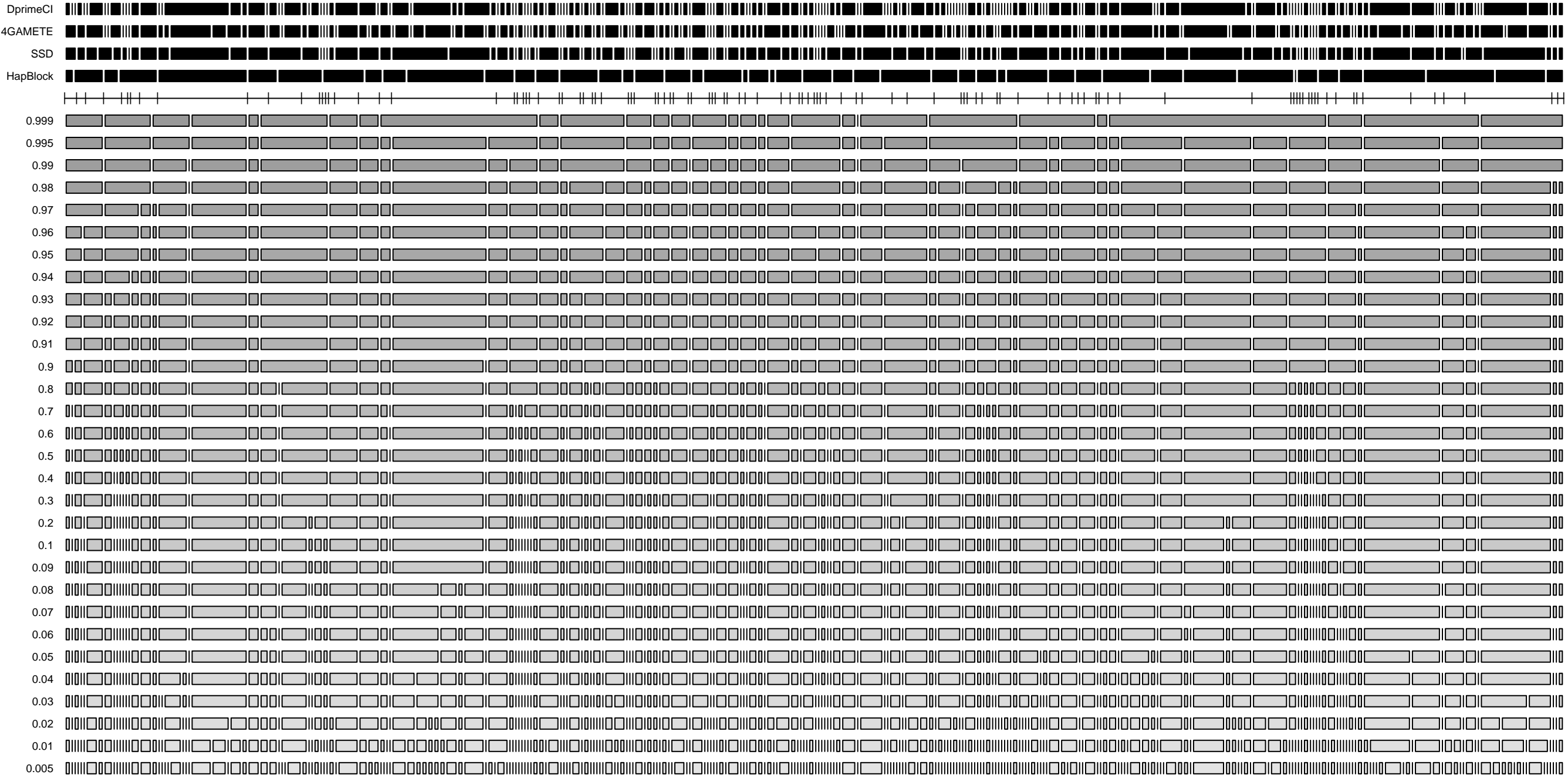

Supplement: Additional file 1 — Comparison of the block partitions on a simulated sample of 200 subjects. The method used is indicated on the left. On the fifth, unlabeled line, ticks are at the positions where at least three of the four methods above it agreed. MATILDE block structures are reported at different probability cutoffs. [file 1471-2164-9-405-S1.pdf]

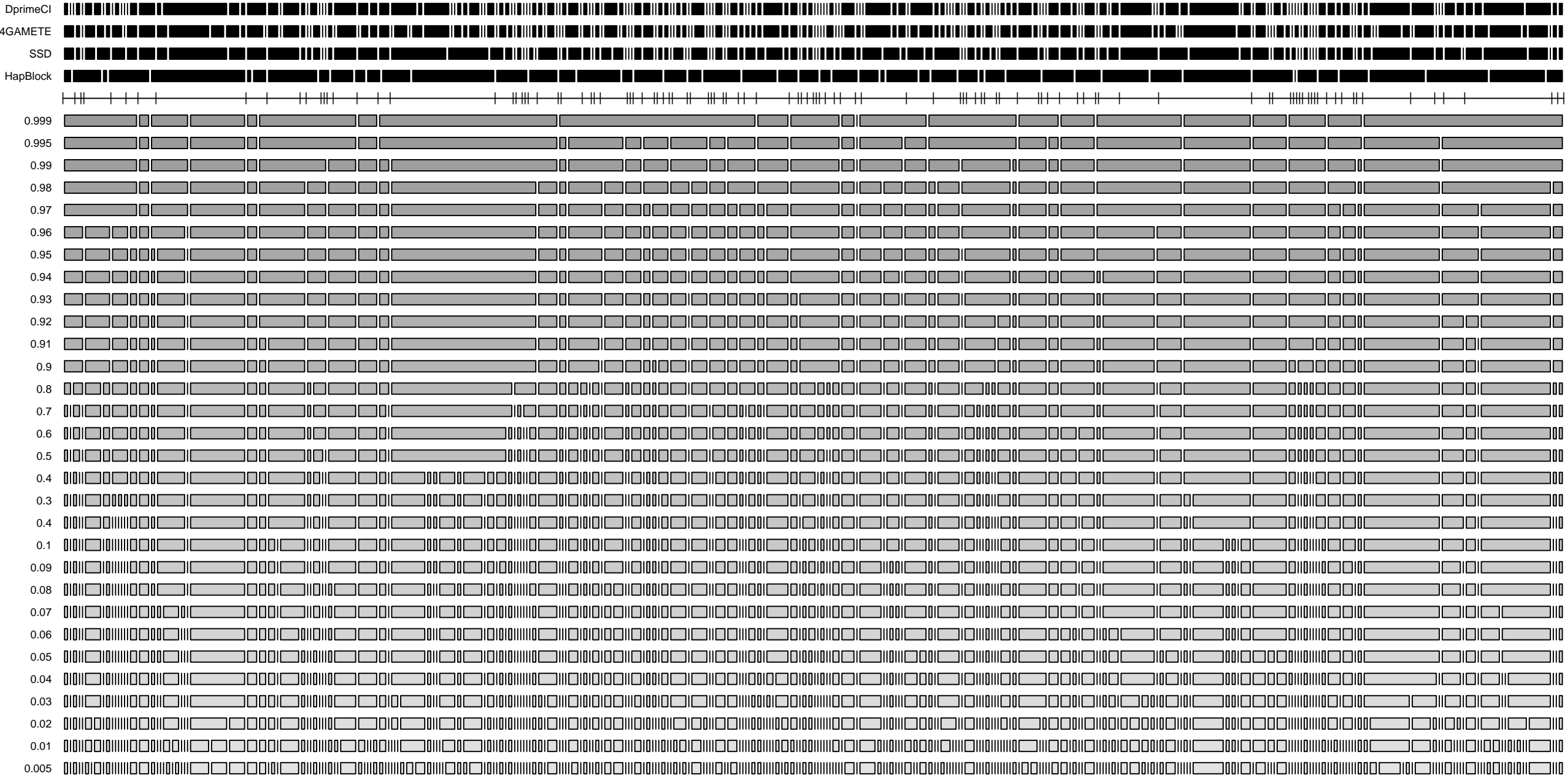

Supplement: Additional file 2 — Comparison of the block partitions on a simulated sample of 400 subjects. The method used is indicated on the left. On the fifth, unlabeled line, ticks are at the positions where at least three of the four methods above it agreed. MATILDE block structures are reported at different probability cutoffs. [file 1471-2164-9-405-S2.pdf]

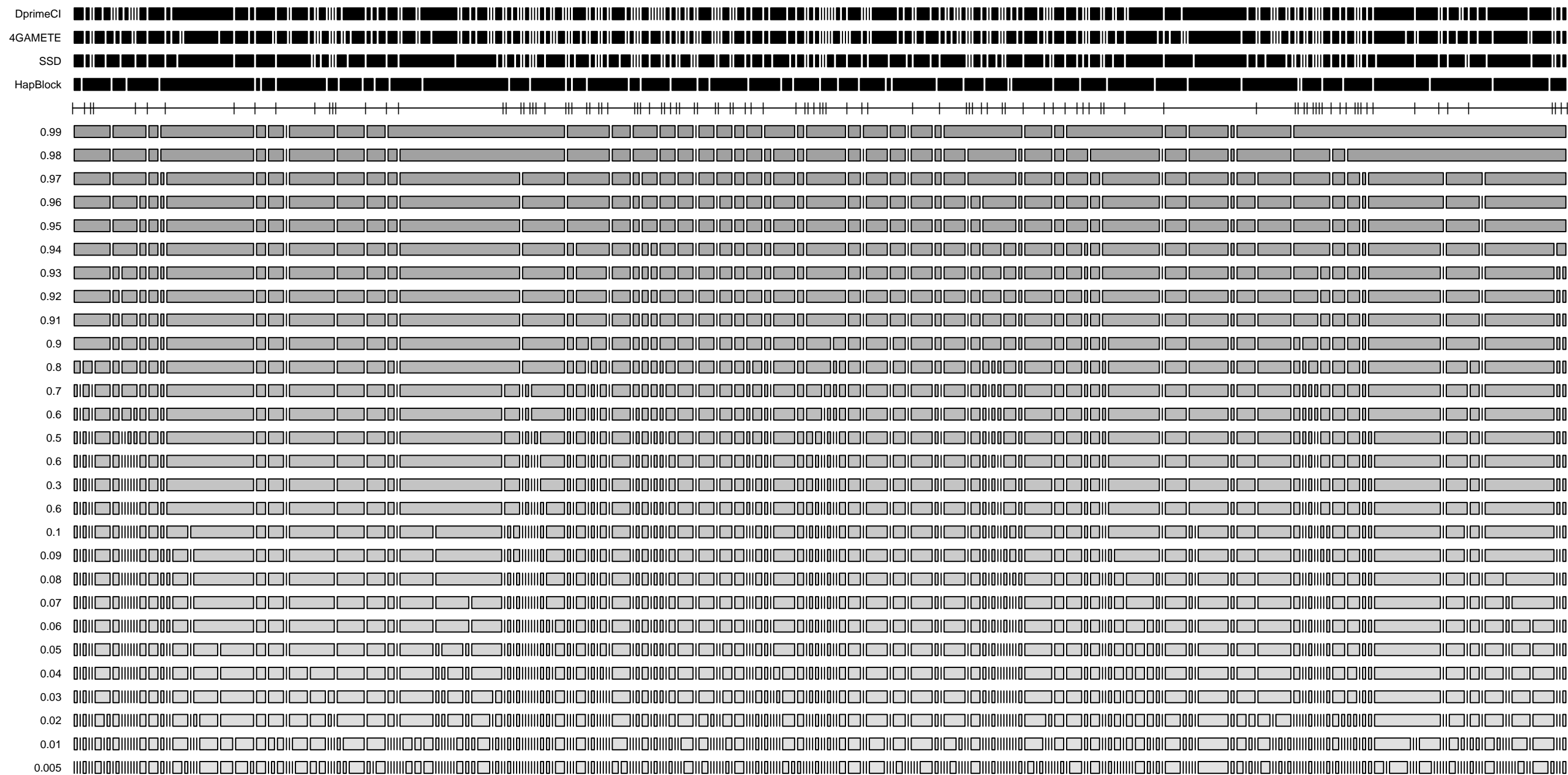

Supplement: Additional file 3 — Comparison of the block partitions on a simulated sample of 600 subjects. The method used is indicated on the left. On the fifth, unlabeled line, ticks are at the positions where at least three of the four methods above it agreed. MATILDE block structures are reported at different probability cutoffs. [file 1471-2164-9-405-S3.pdf]

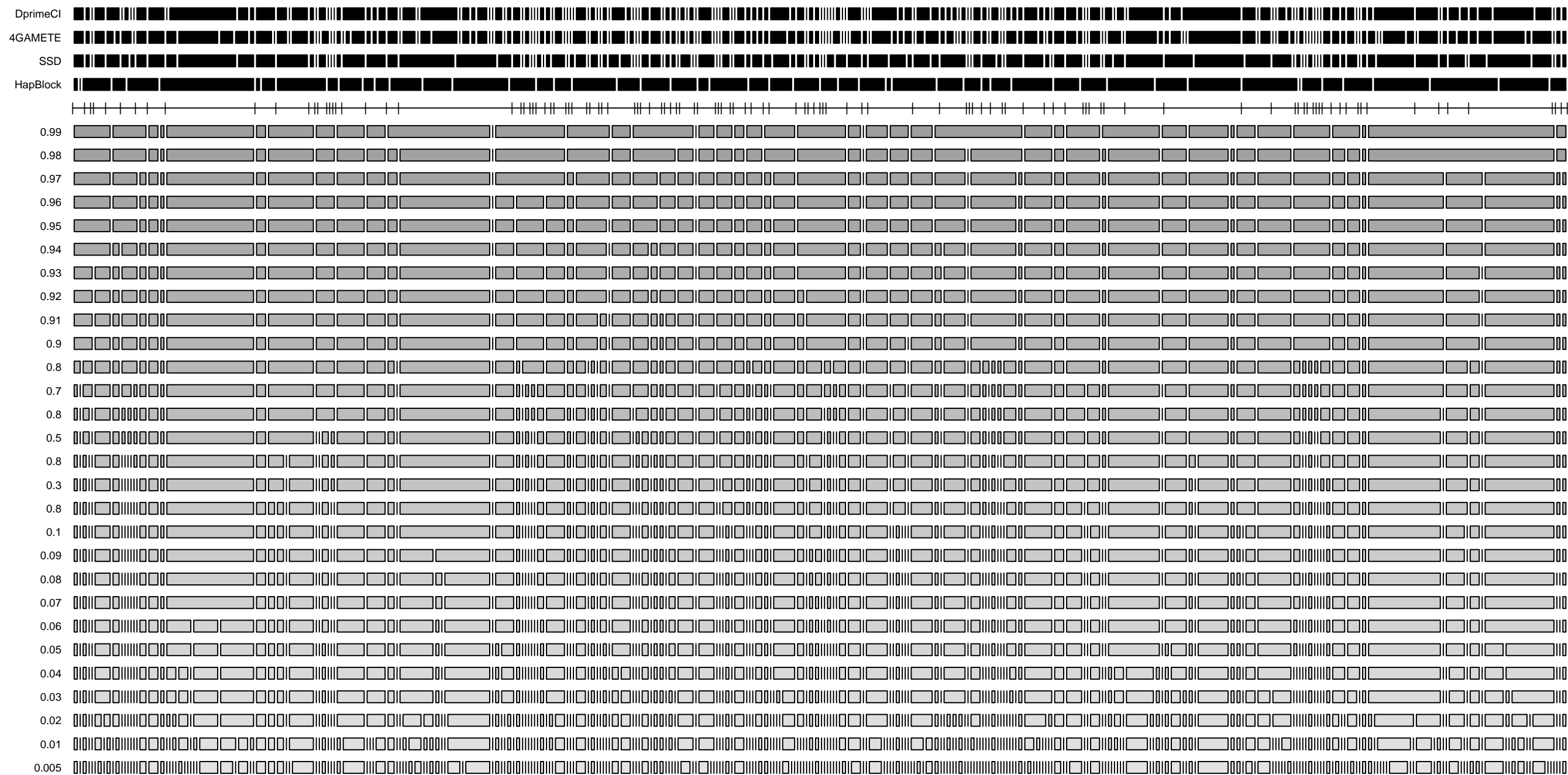

Supplement: Additional file 4 — Comparison of the block partitions on a simulated sample of 800 subjects. The method used is indicated on the left. On the fifth, unlabeled line, ticks are at the positions where at least three of the four methods above it agreed. MATILDE block structures are reported at different probability cutoffs. [file 1471-2164-9-405-S4.pdf]

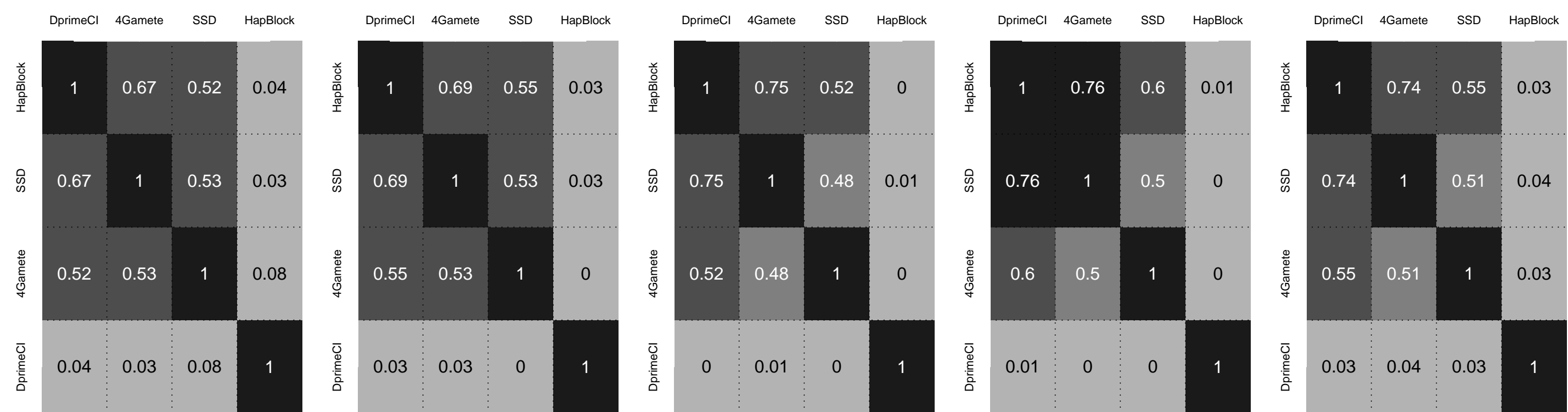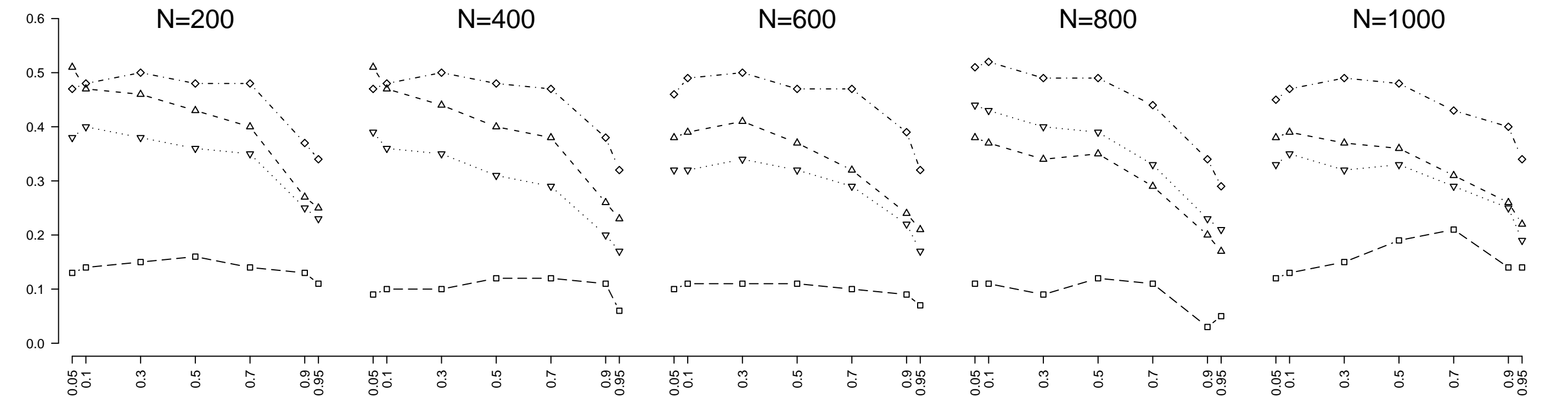

Supplement: Additional file 5 — Agreement between all block partitioning methods. Upper panels: pairwise κ s between the four most common methods, by sample size. Lower panel: κ s between MATILDE and the four most common methods, by probability cutoff (x-axis) and sample size. Symbols: triangle = DprimeCI, diamond = SSD, reverse triangle = 4Gamete, and square = HapBlock. [file 1471-2164-9-405-S5.pdf]

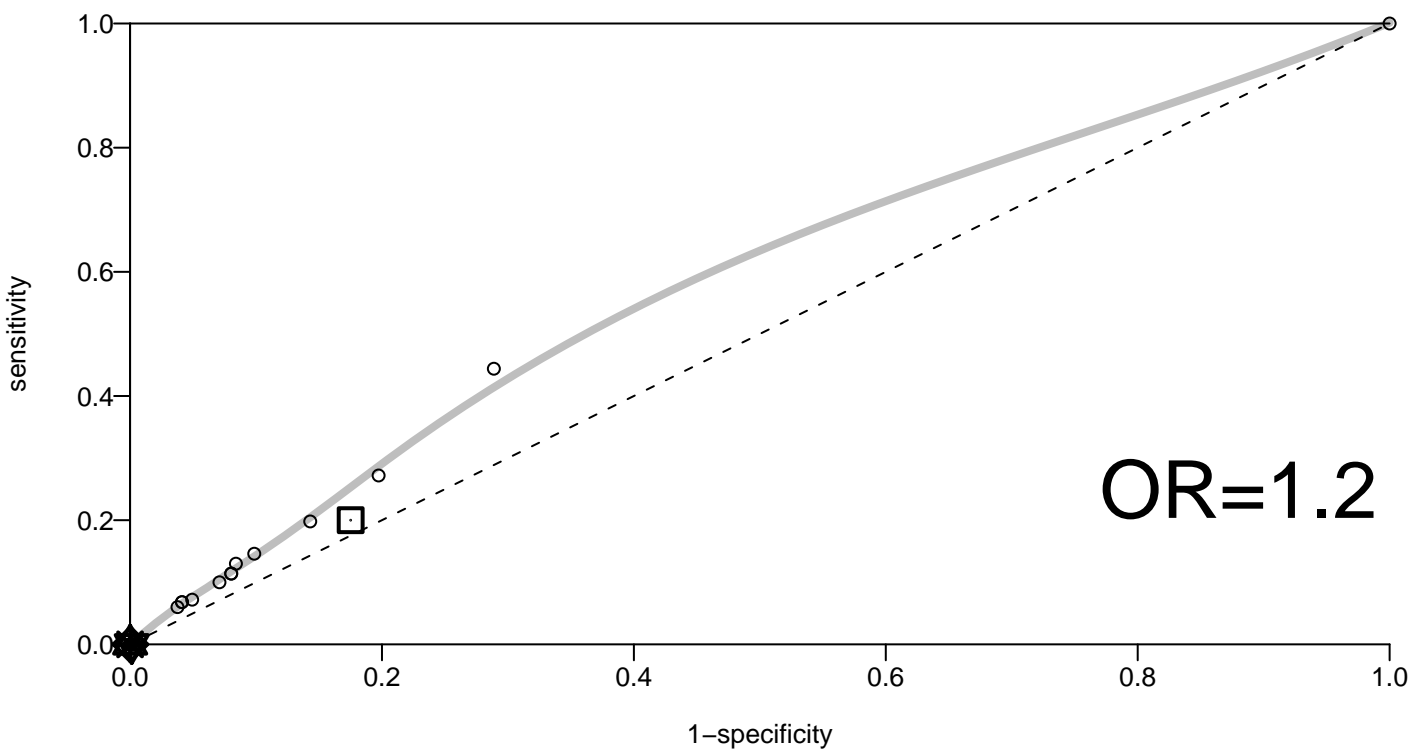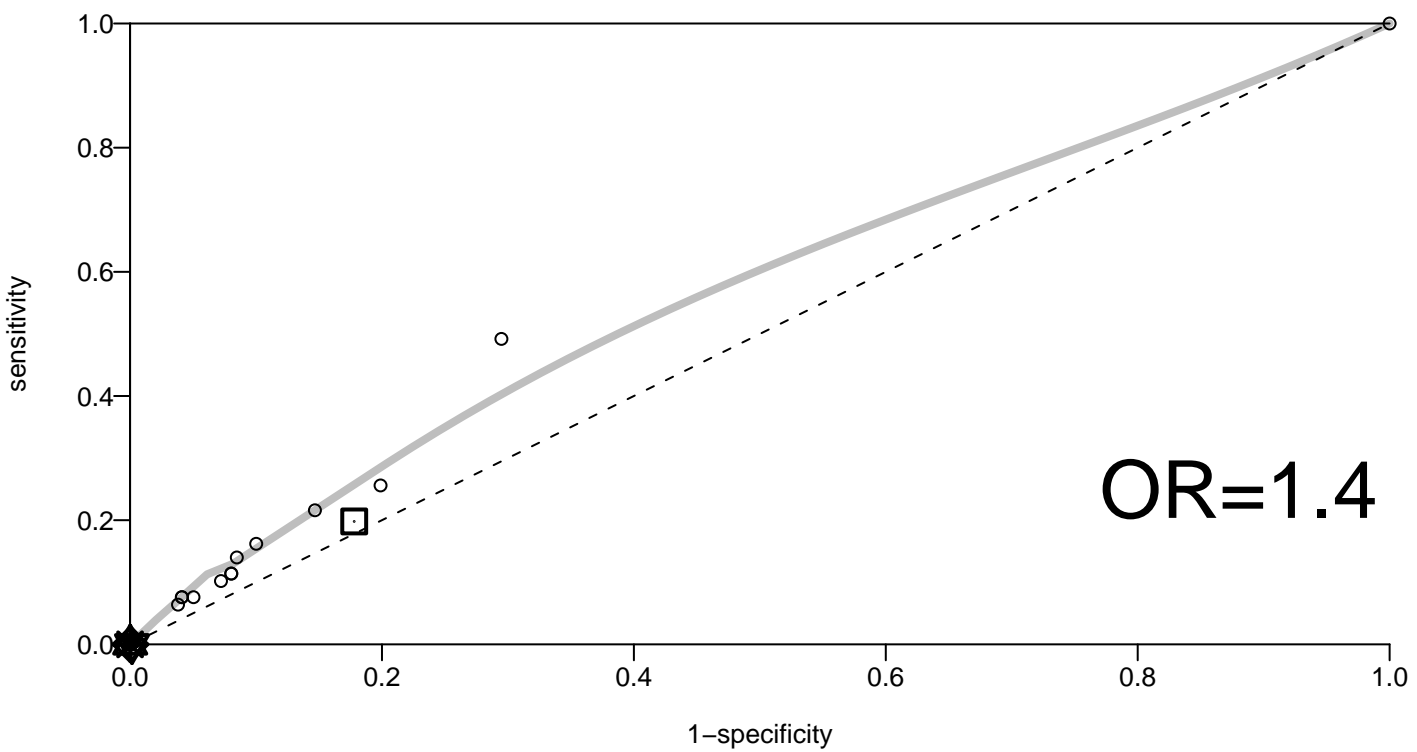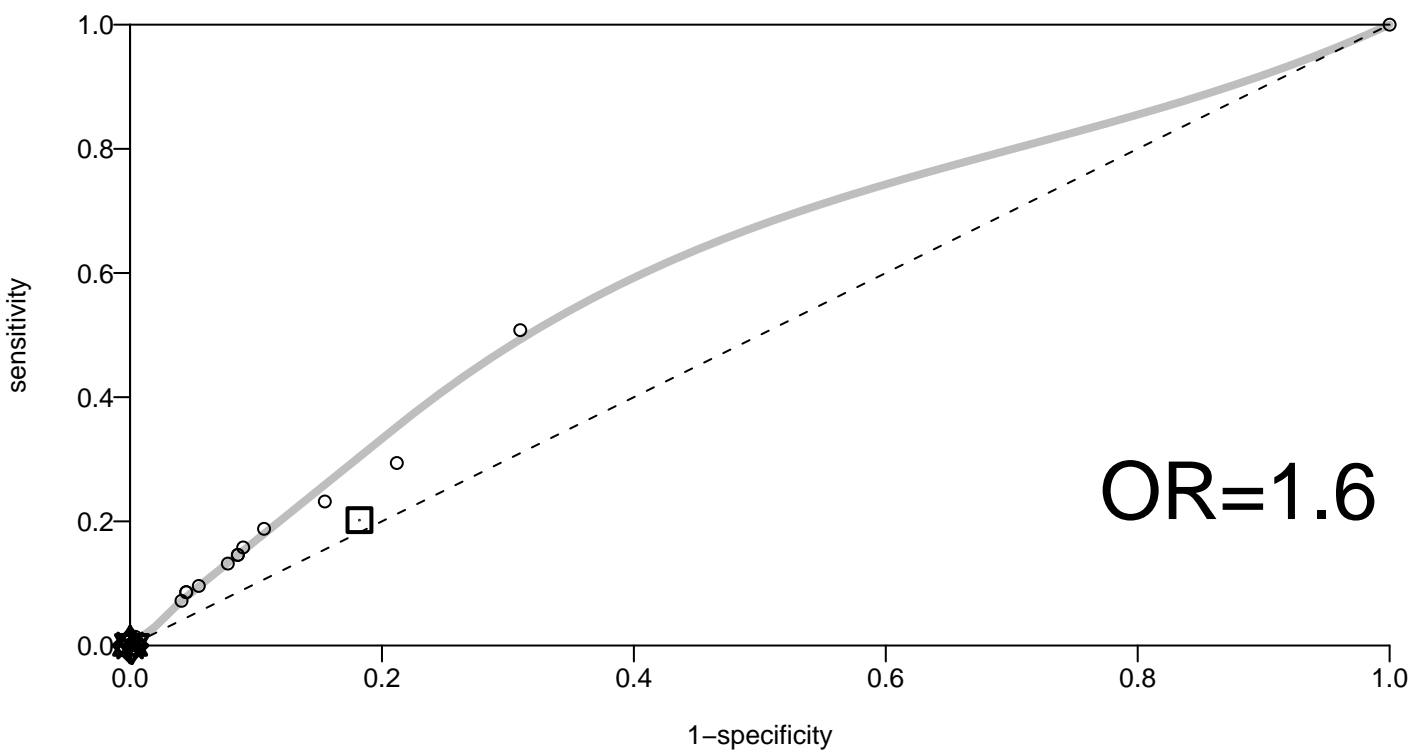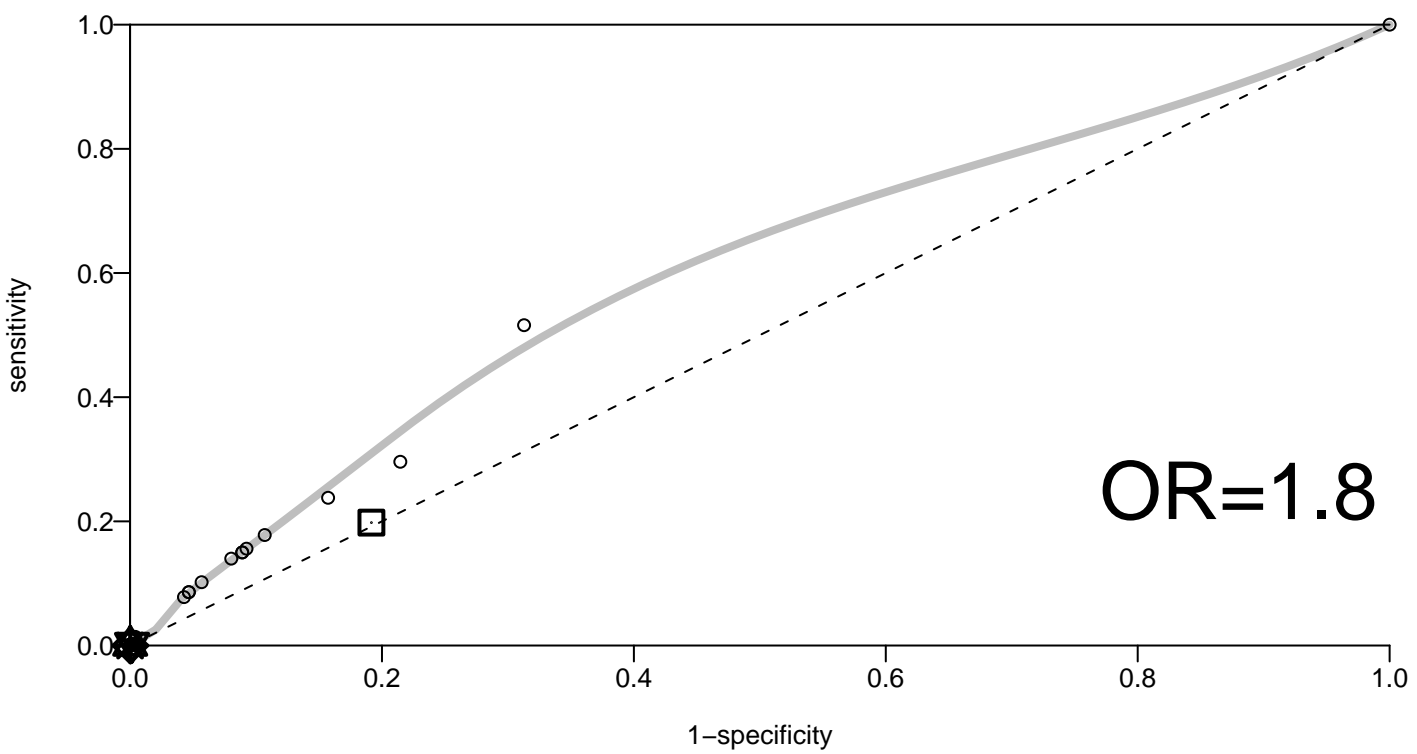

Supplement: Additional file 6 — Comparison of methods' sensitivity and specificity. Data refer to the simulation of 100 cases and 100 controls assuming a dominant model. Each panel reports the sensitivity/specificity tradeoff for DIprimeCI (triangle), 4Gamete (reversed triangle), the SSD (diamond), HapBlock (square) and MATILDE (represented by points on the ROC curves, graphed as circles, and a smooth estimate of the ROC curve). In addition an allele-based single-SNP association analysis is represented by an "x" while a genotype-based single-SNP association analysis is represented by a "+". Four effect sizes were considered: the OR is 1.2, 1.4, 1.6 and 1.8, respectively. [file 1471-2164-9-405-S6.pdf]

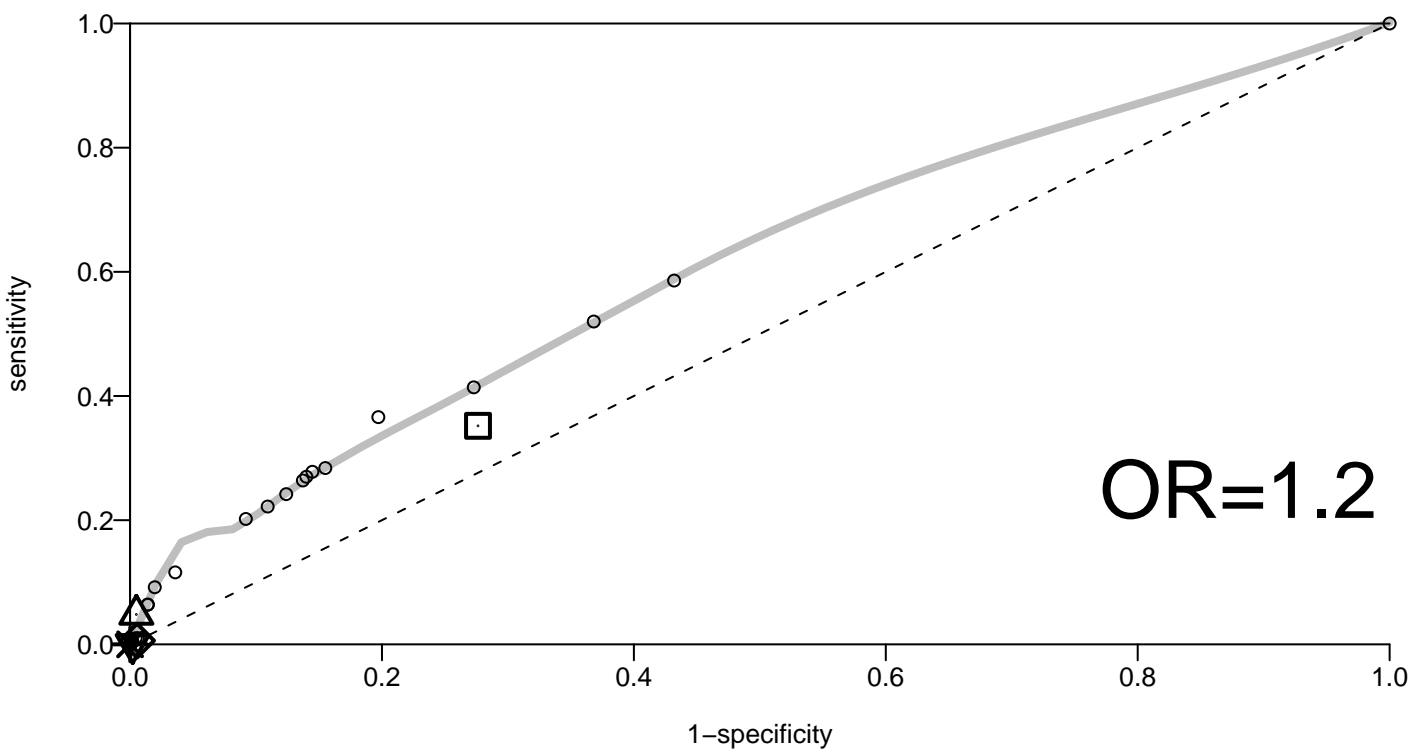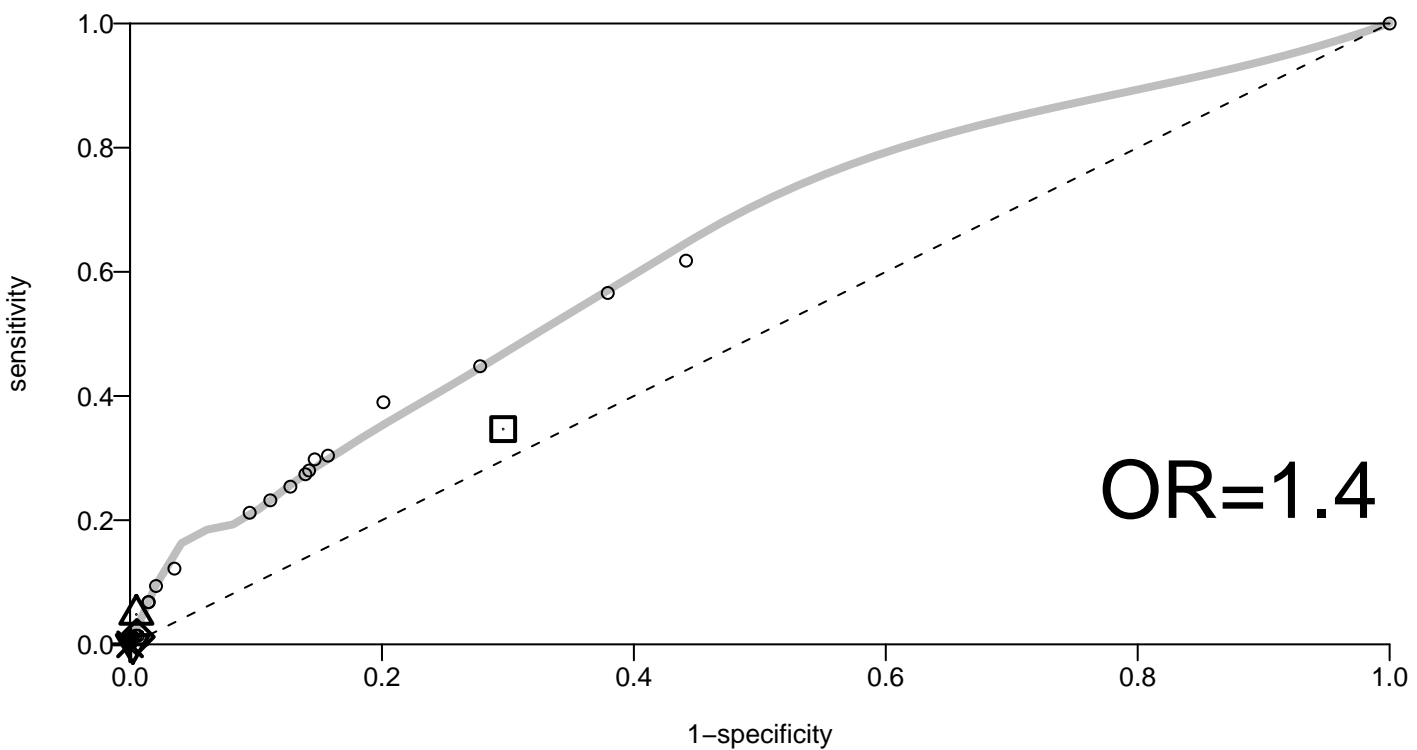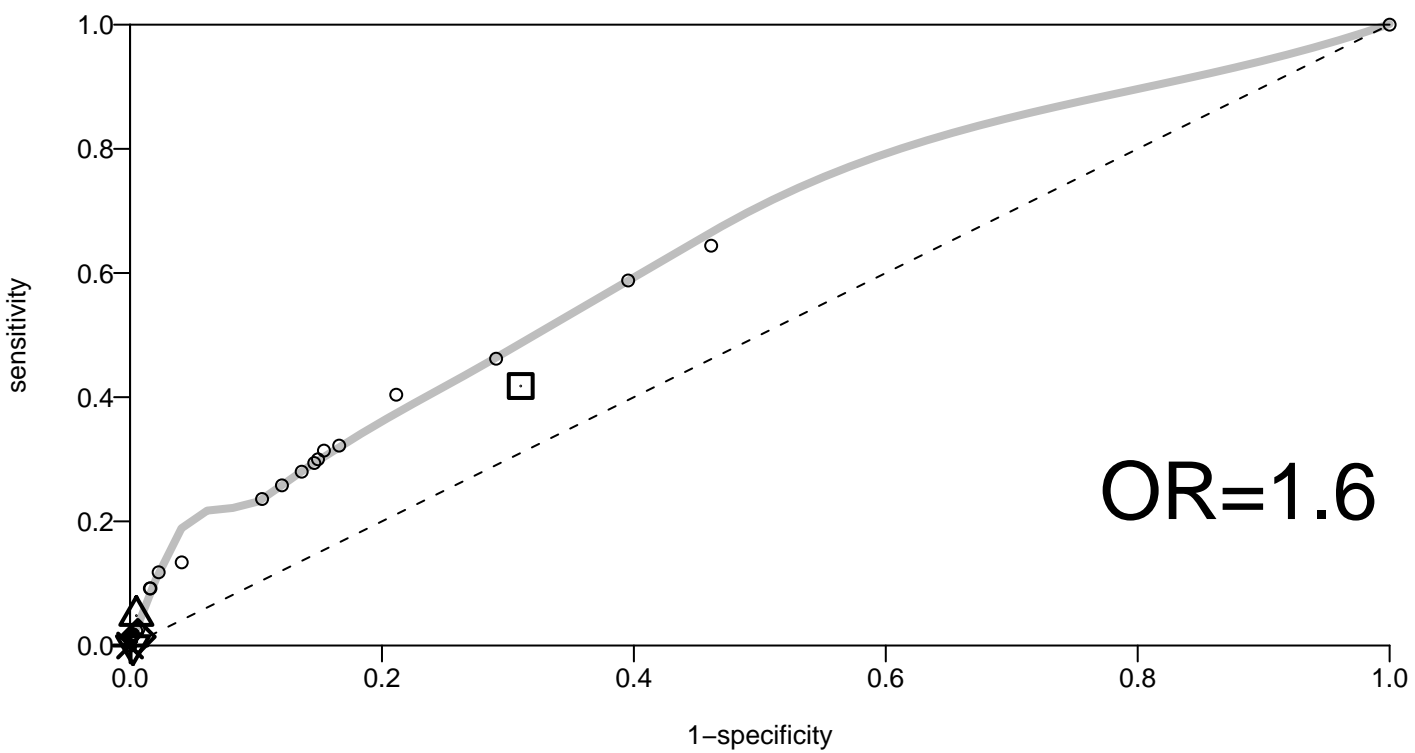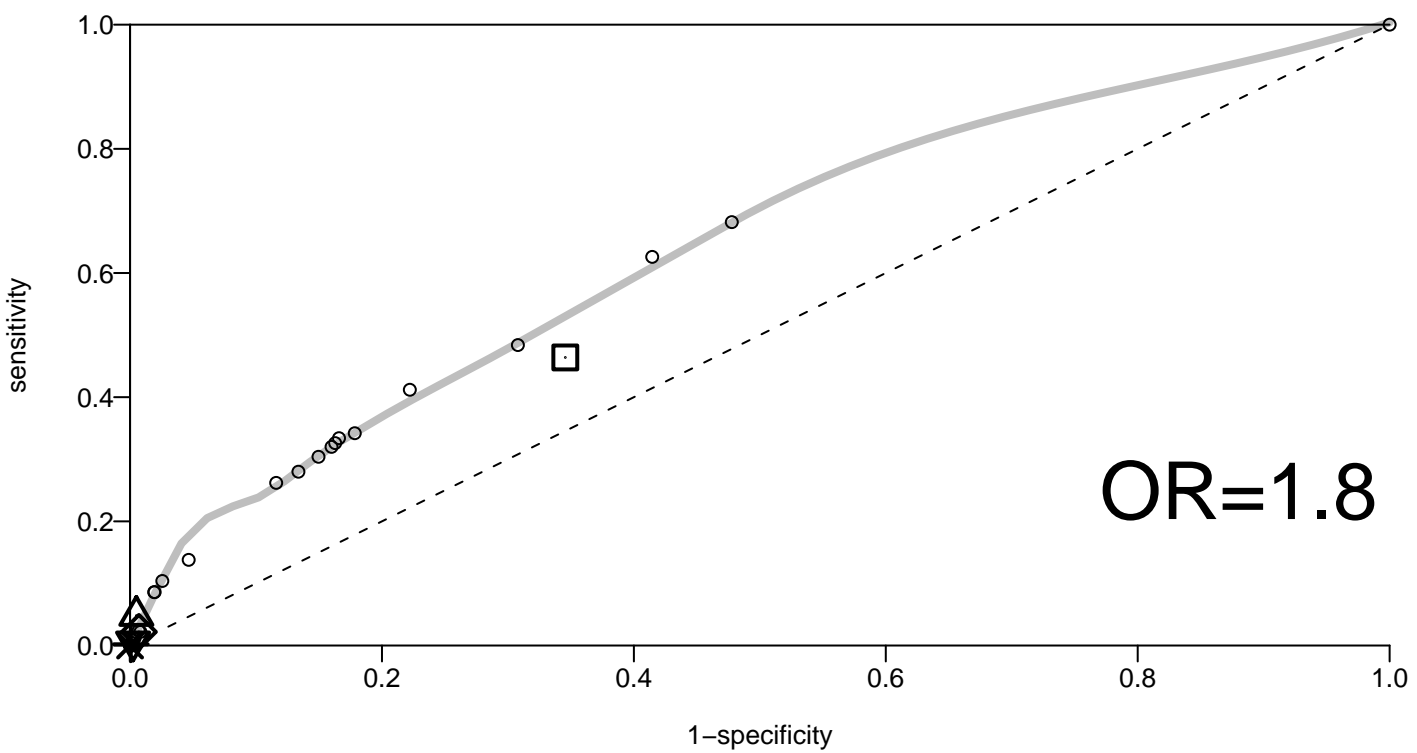

Supplement: Additional file 7 — Comparison of methods' sensitivity and specificity. Data refer to the simulation of 200 cases and 200 controls assuming a dominant model. Each panel reports the sensitivity/specificity tradeoff for DIprimeCI (triangle), 4Gamete (reversed triangle), the SSD (diamond), HapBlock (square) and MATILDE (represented by points on the ROC curves, graphed as circles, and a smooth estimate of the ROC curve). In addition an allele-based single-SNP association analysis is represented by an "x" while a genotype-based single-SNP association analysis is represented by a "+". Four effect sizes were considered: the OR is 1.2, 1.4, 1.6 and 1.8, respectively. [file 1471-2164-9-405-S7.pdf]

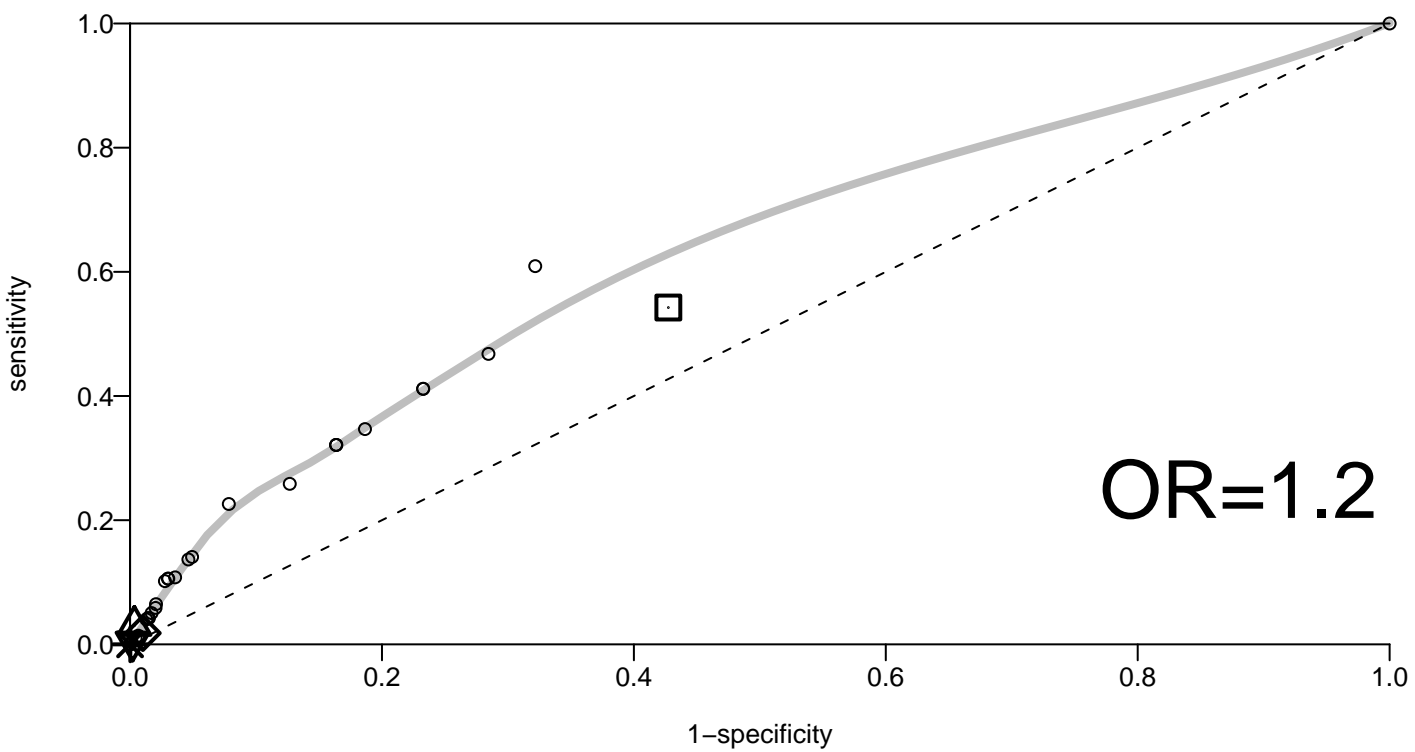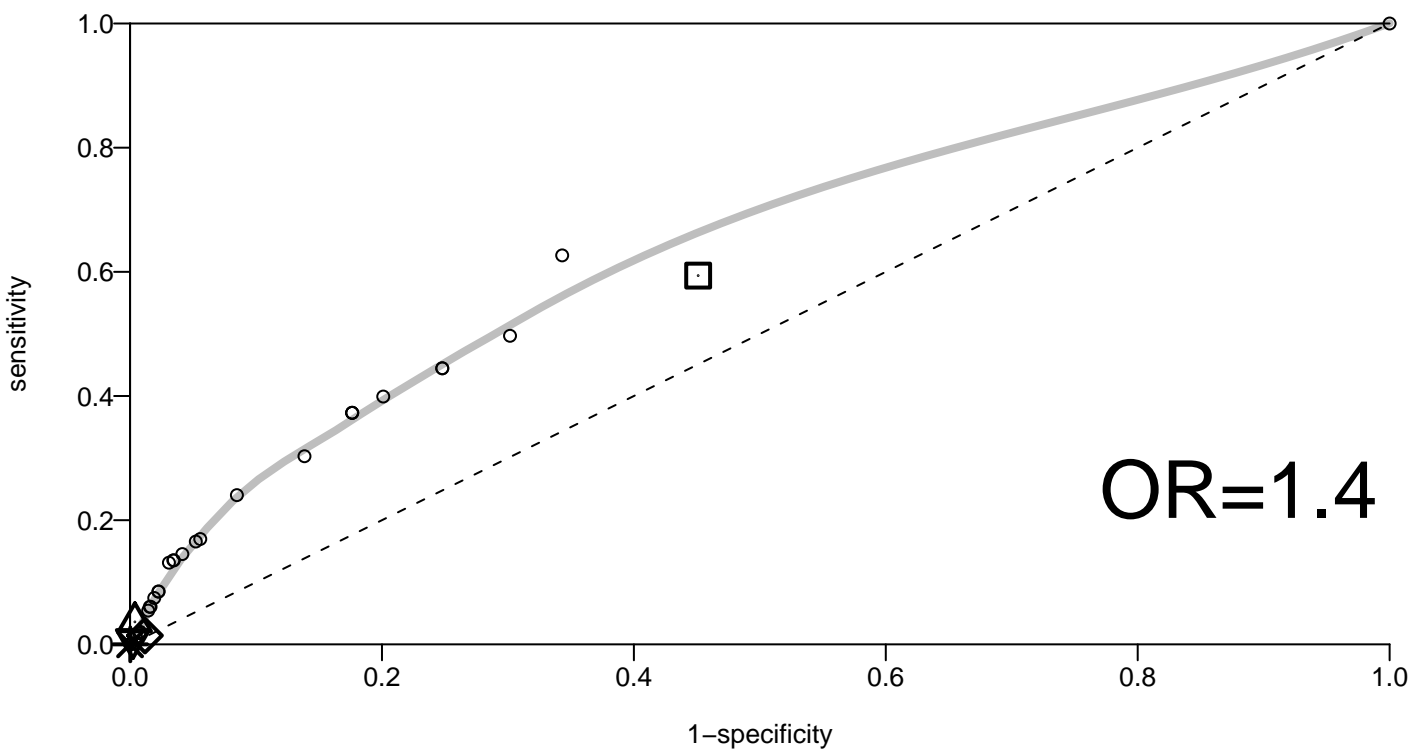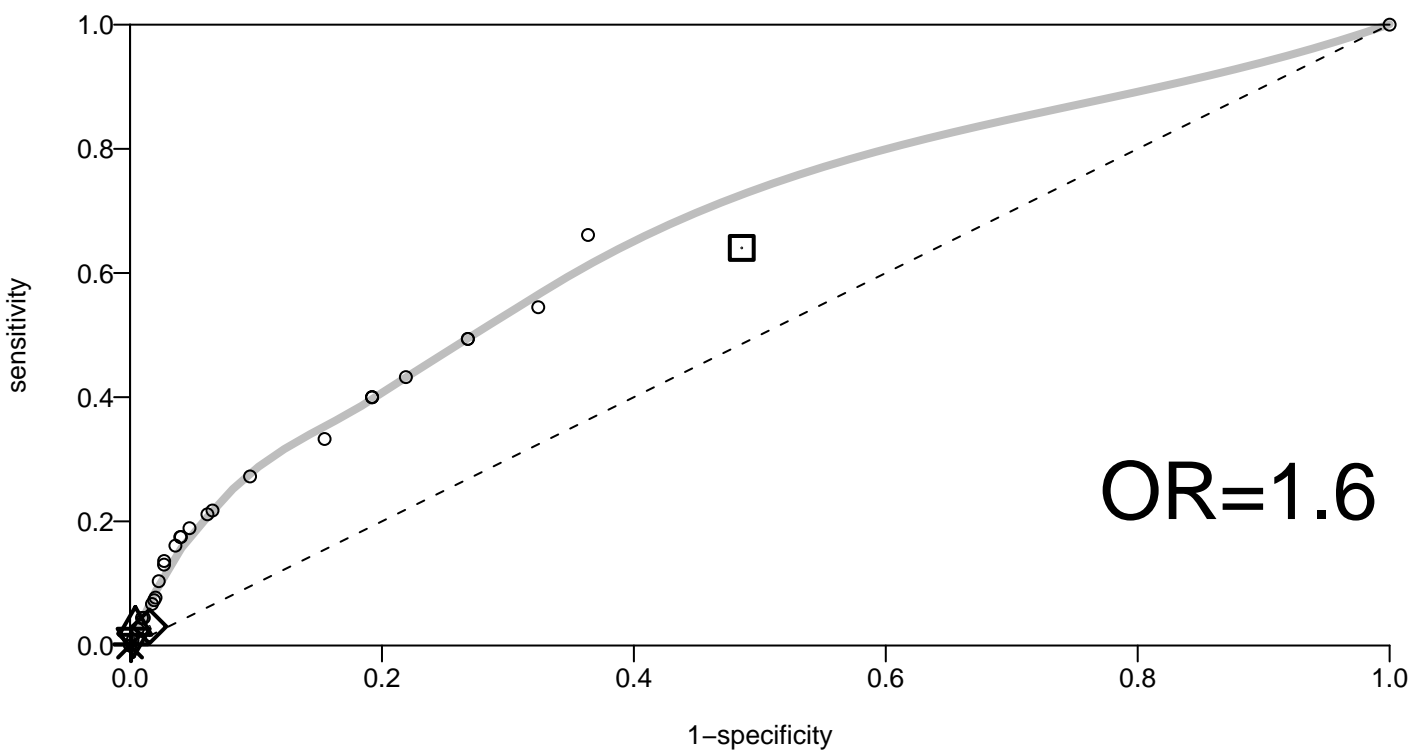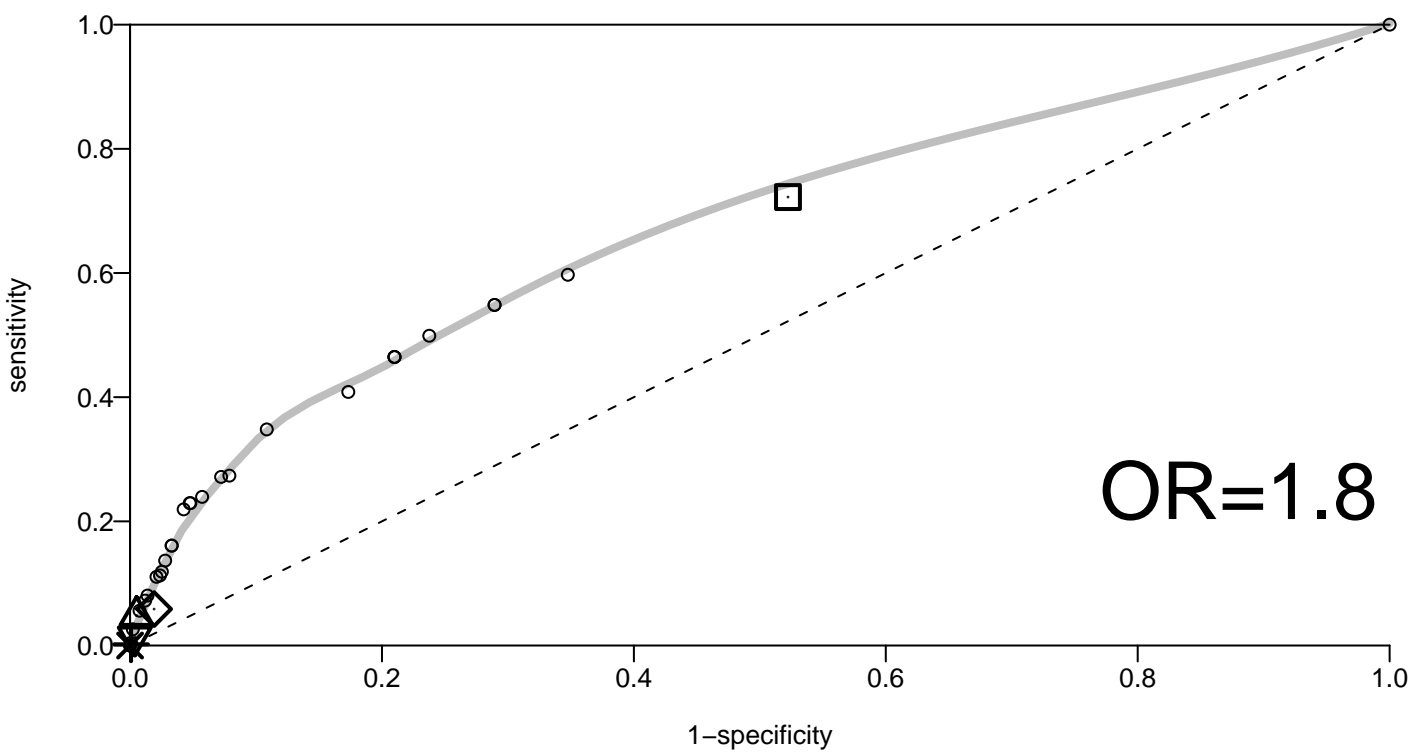

Supplement: Additional file 8 — Comparison of methods' sensitivity and specificity. Data refer to the simulation of 300 cases and 300 controls assuming a dominant model. Each panel reports the sensitivity/specificity tradeoff for DIprimeCI (triangle), 4Gamete (reversed triangle), the SSD (diamond), HapBlock (square) and MATILDE (represented by points on the ROC curves, graphed as circles, and a smooth estimate of the ROC curve). In addition an allele-based single-SNP association analysis is represented by an "x" while a genotype-based single-SNP association analysis is represented by a "+". Four effect sizes were considered: the OR is 1.2, 1.4, 1.6 and 1.8, respectively. [file 1471-2164-9-405-S8.pdf]

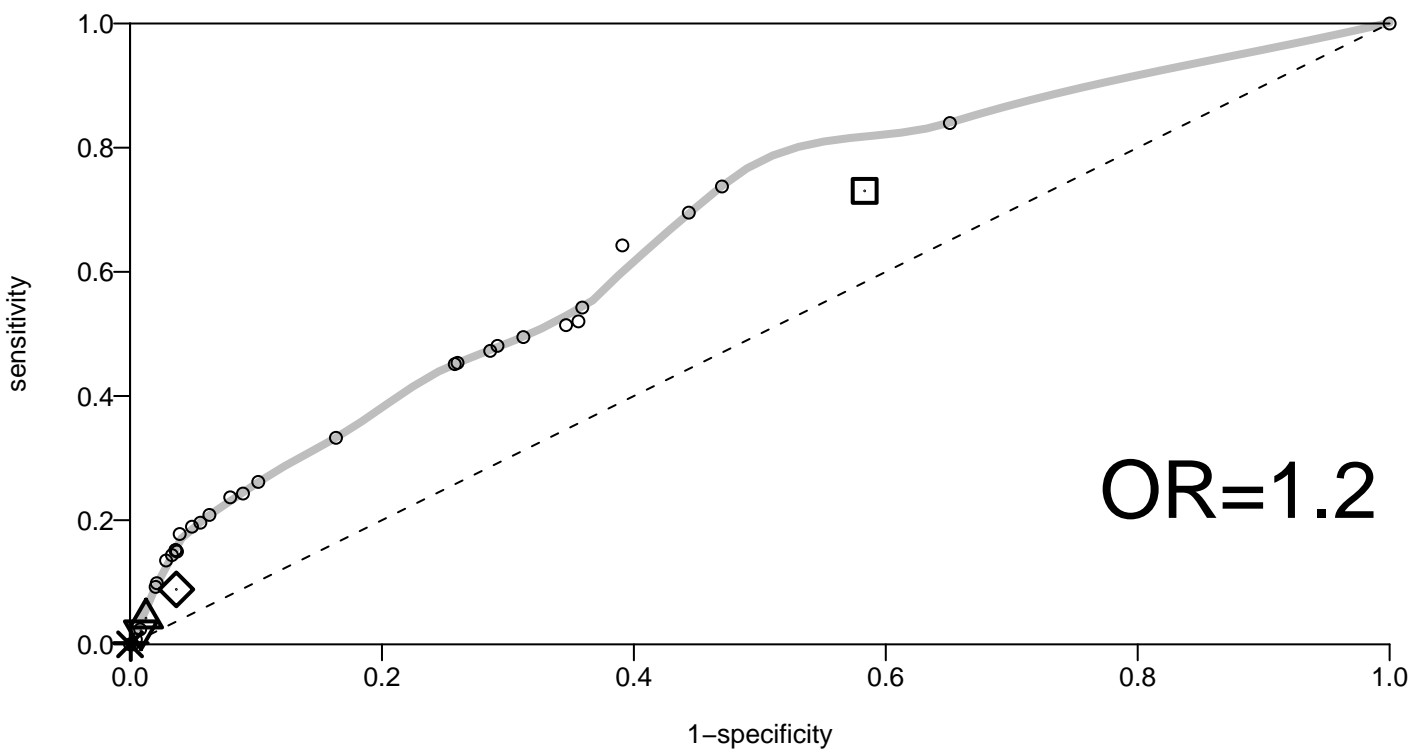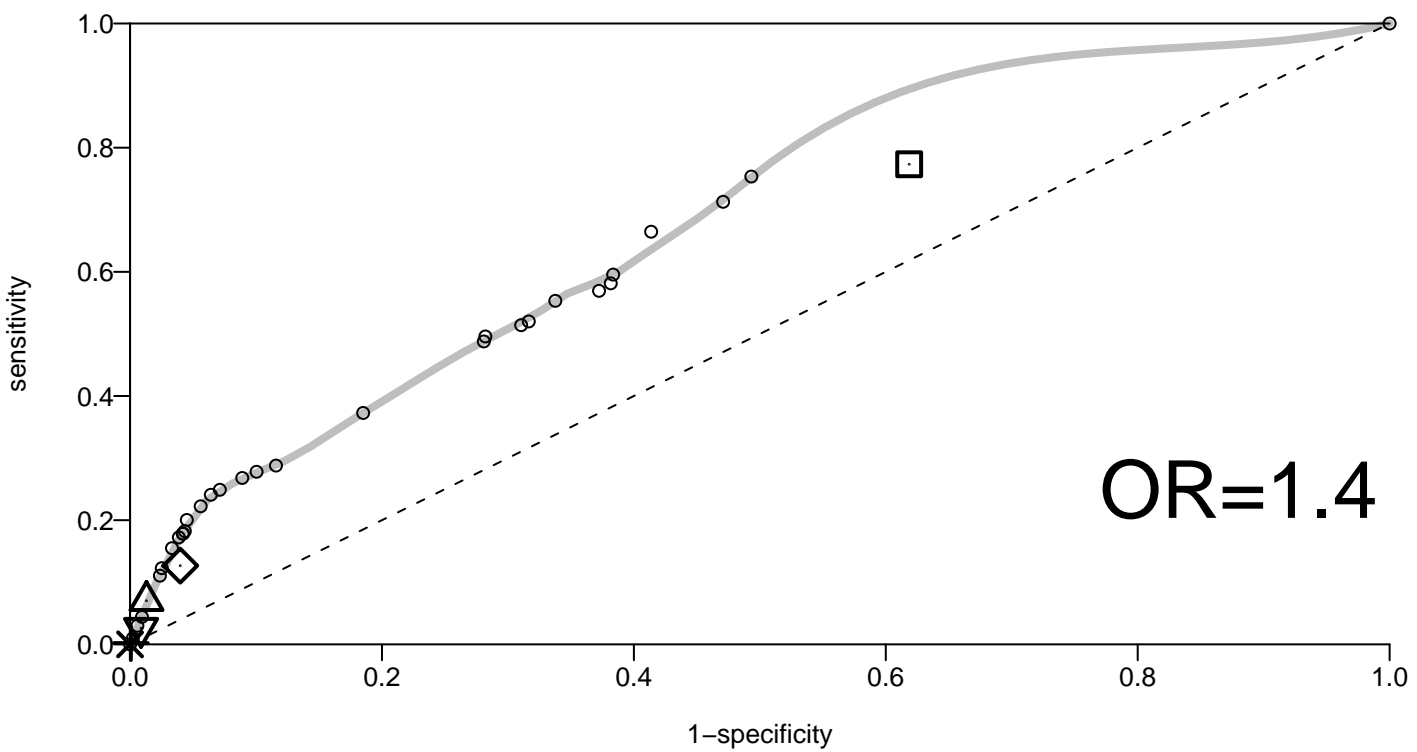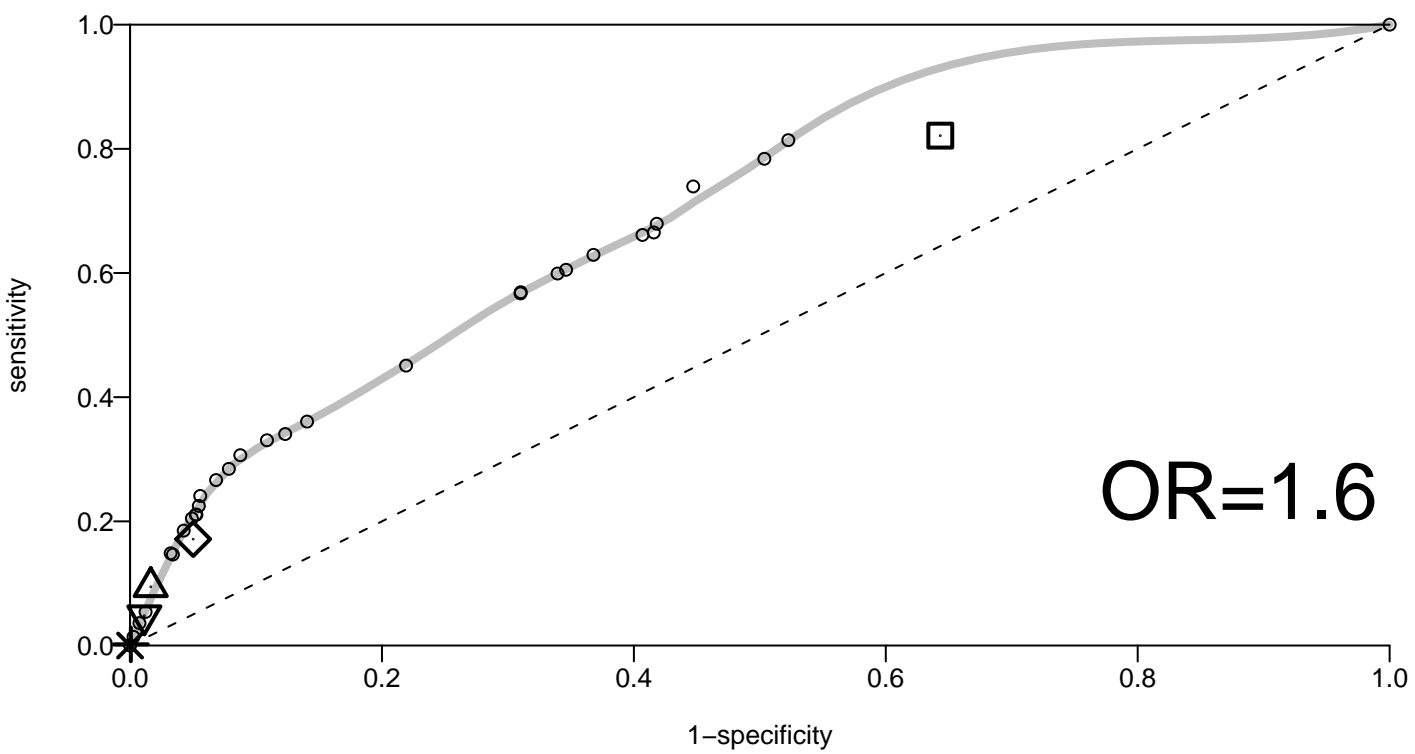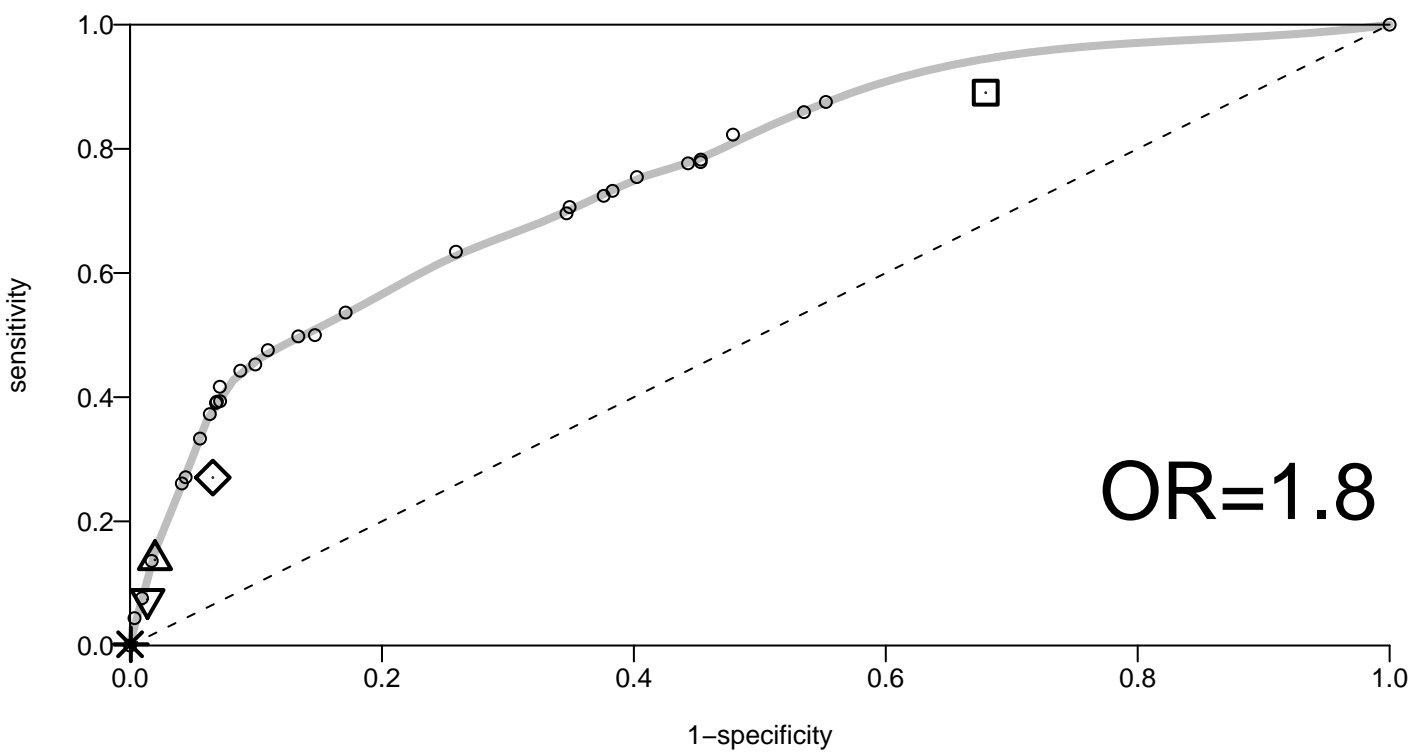

Supplement: Additional file 9 — Comparison of methods' sensitivity and specificity. Data refer to the simulation of 400 cases and 400 controls assuming a dominant model. Each panel reports the sensitivity/specificity tradeoff for DIprimeCI (triangle), 4Gamete (reversed triangle), the SSD (diamond), HapBlock (square) and MATILDE (represented by points on the ROC curves, graphed as circles, and a smooth estimate of the ROC curve). In addition an allele-based single-SNP association analysis is represented by an "x" while a genotype-based single-SNP association analysis is represented by a "+". Four effect sizes were considered: the OR is 1.2, 1.4, 1.6 and 1.8, respectively. [file 1471-2164-9-405-S9.pdf]

OR=1.2

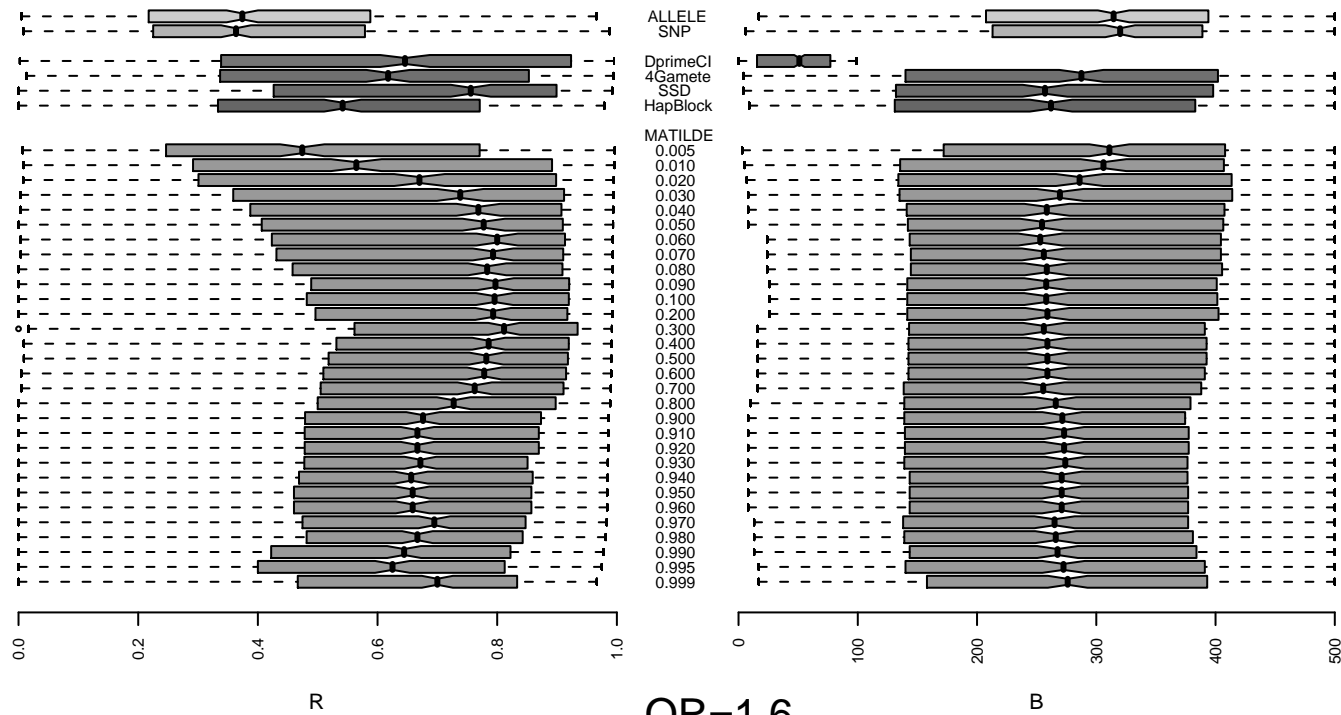

OR=1.4

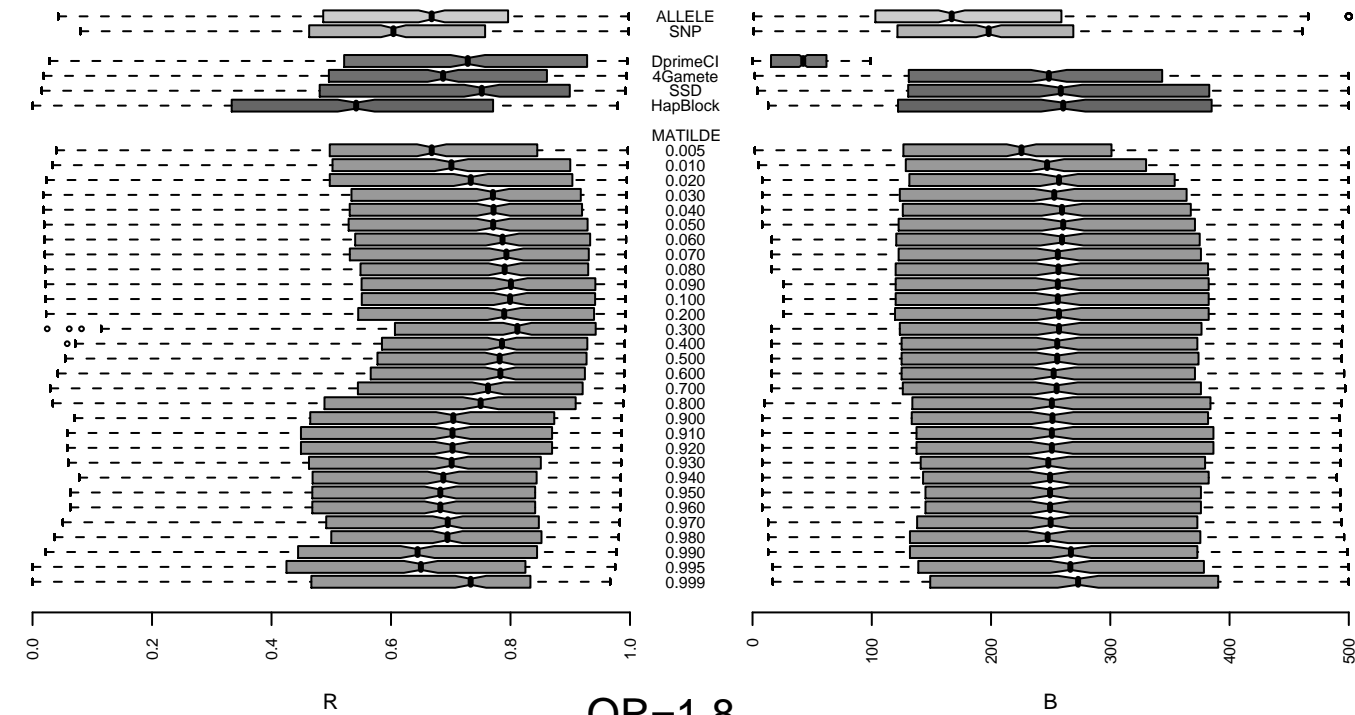

OR=1.6

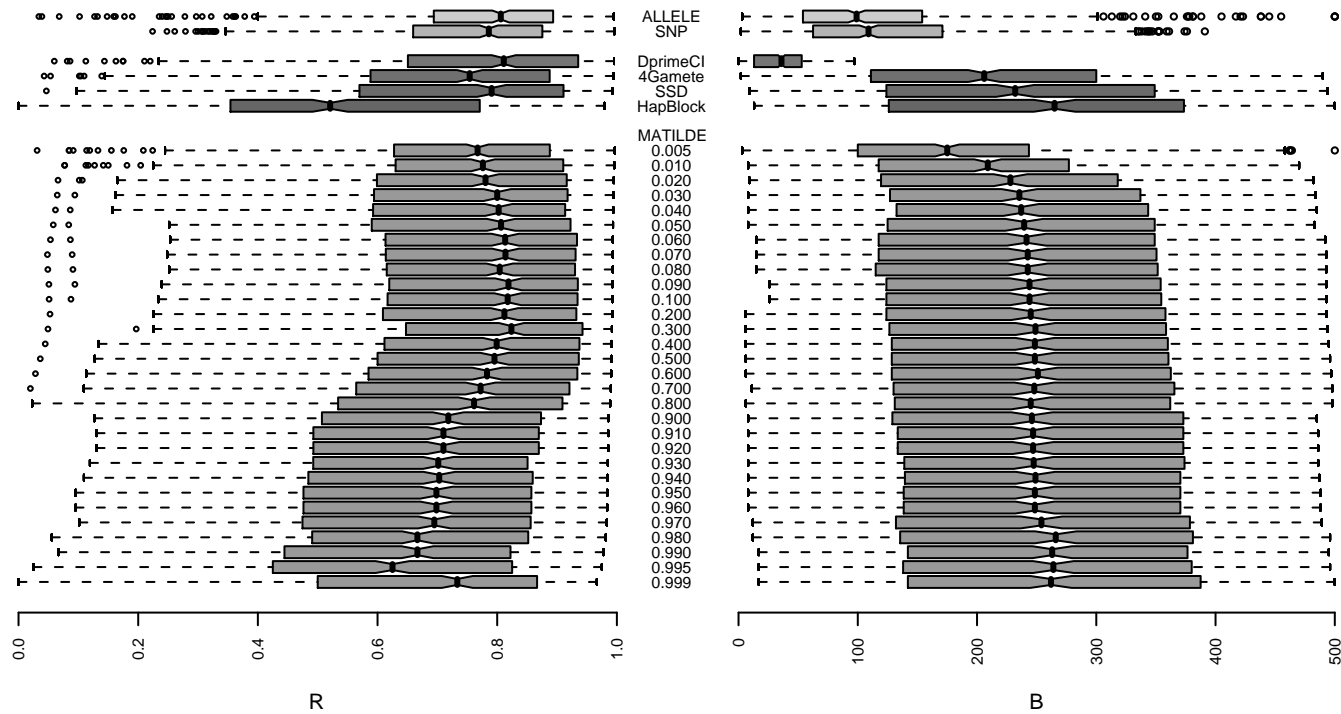

OR=1.8

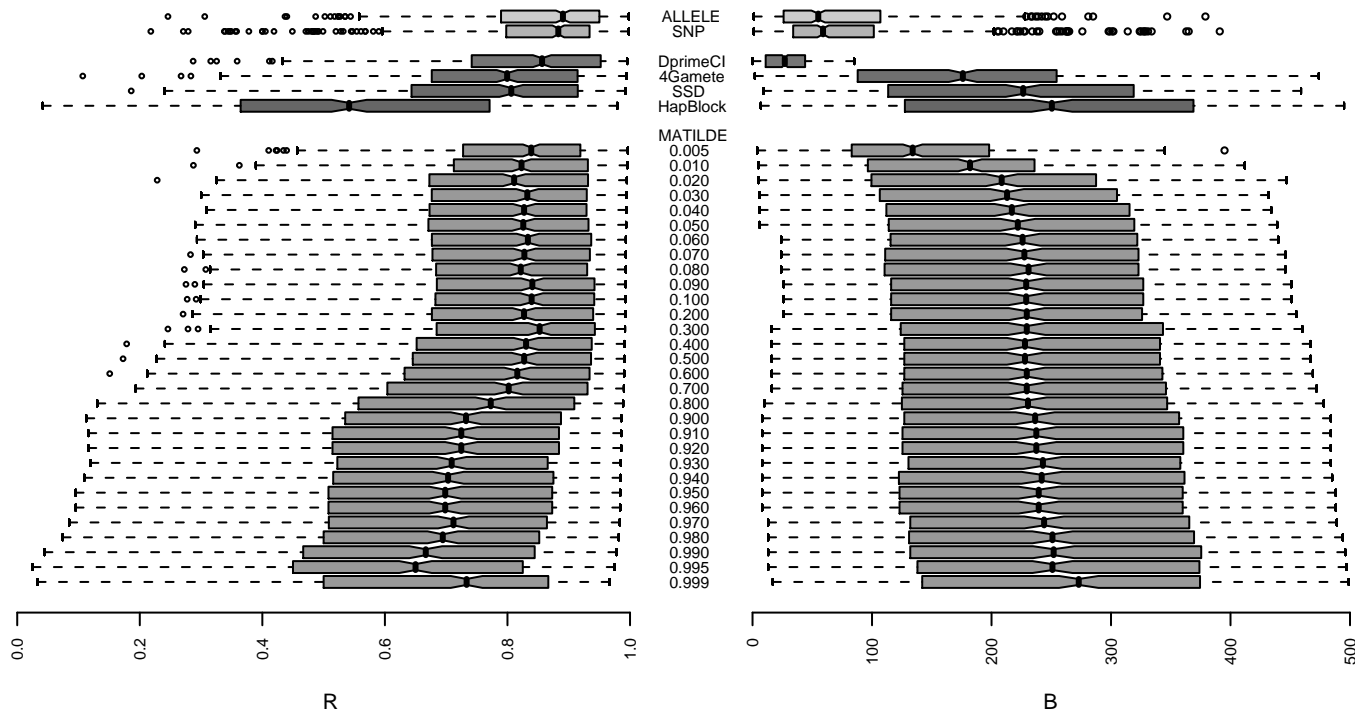

Supplement: Additional file 10 — Parallel distribution of the statistics R (relative position of the block containing the right SNP) and B (number of SNPs belonging to blocks classified not worse than the true SNP) for a sample size of 100 cases and 100 controls. Four effect sizes were considered: the OR is 1.2, 1.4, 1.6 and 1.8, respectively. For each panel, the results of simulation with the allele-based single-SNP method, the genotype-based single-SNP analysis, the four common methods (DprimeCI, 4Gamete, SSD and HapBlock) and the MATILDE at various cutoff thresholds are listed. [file 1471-2164-9-405-S10.pdf]

OR=1.2

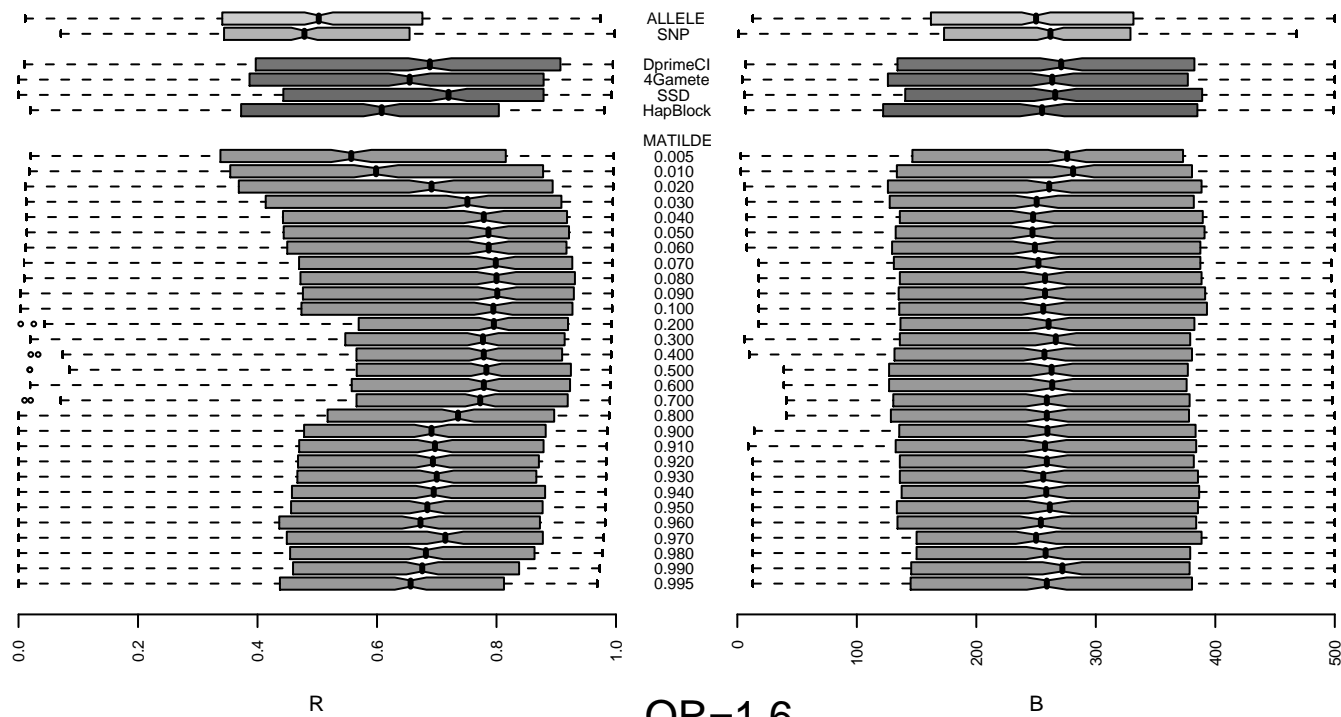

OR=1.4

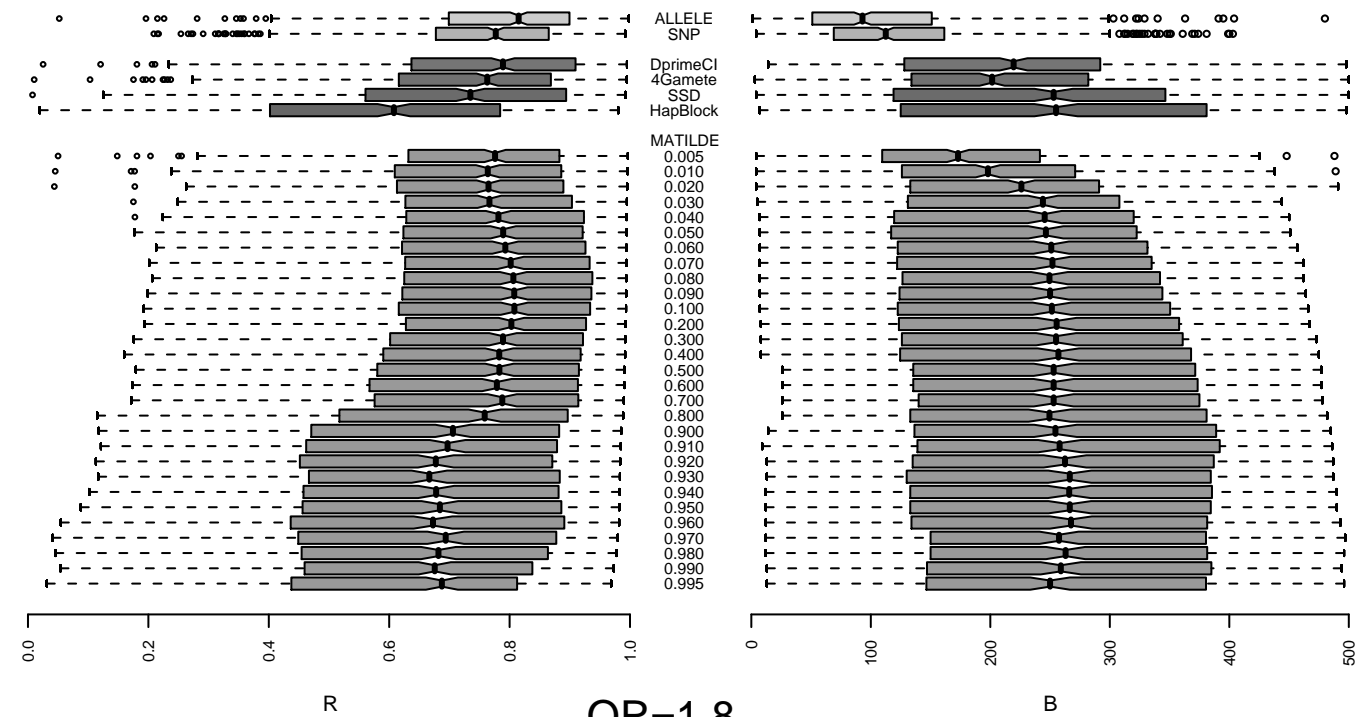

OR=1.6

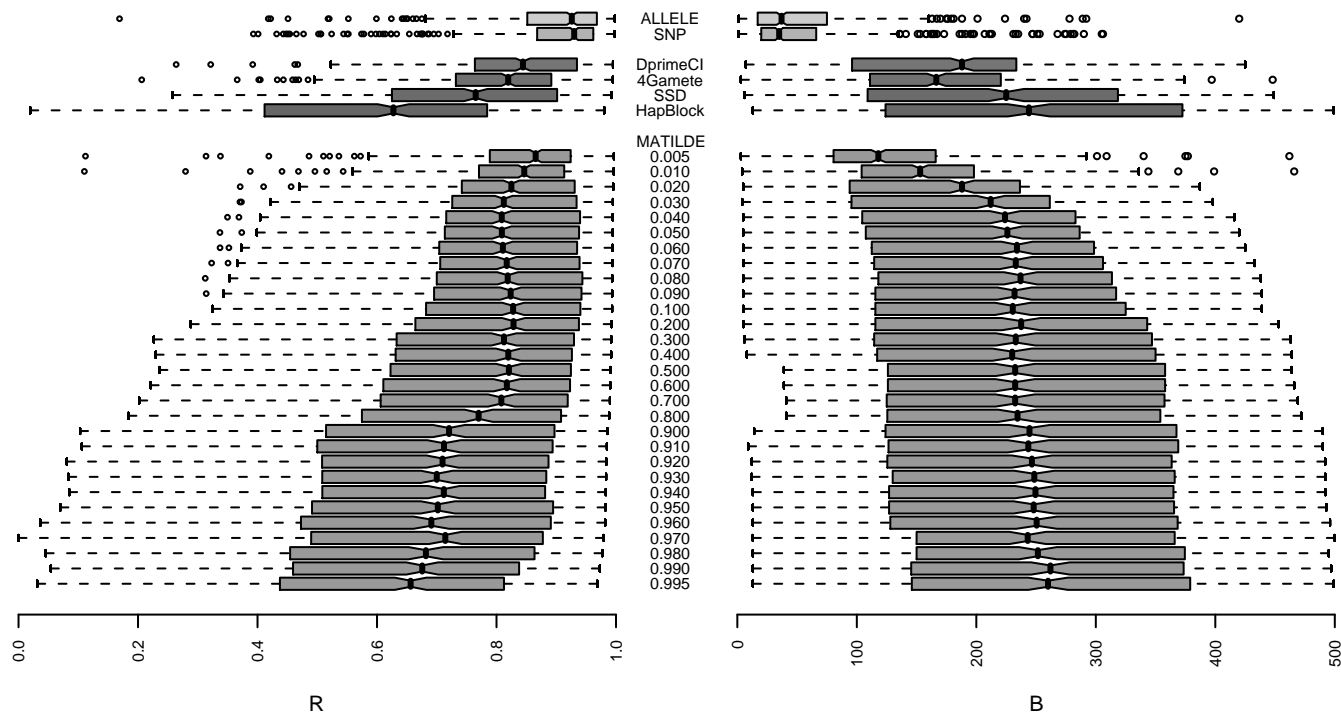

OR=1.8

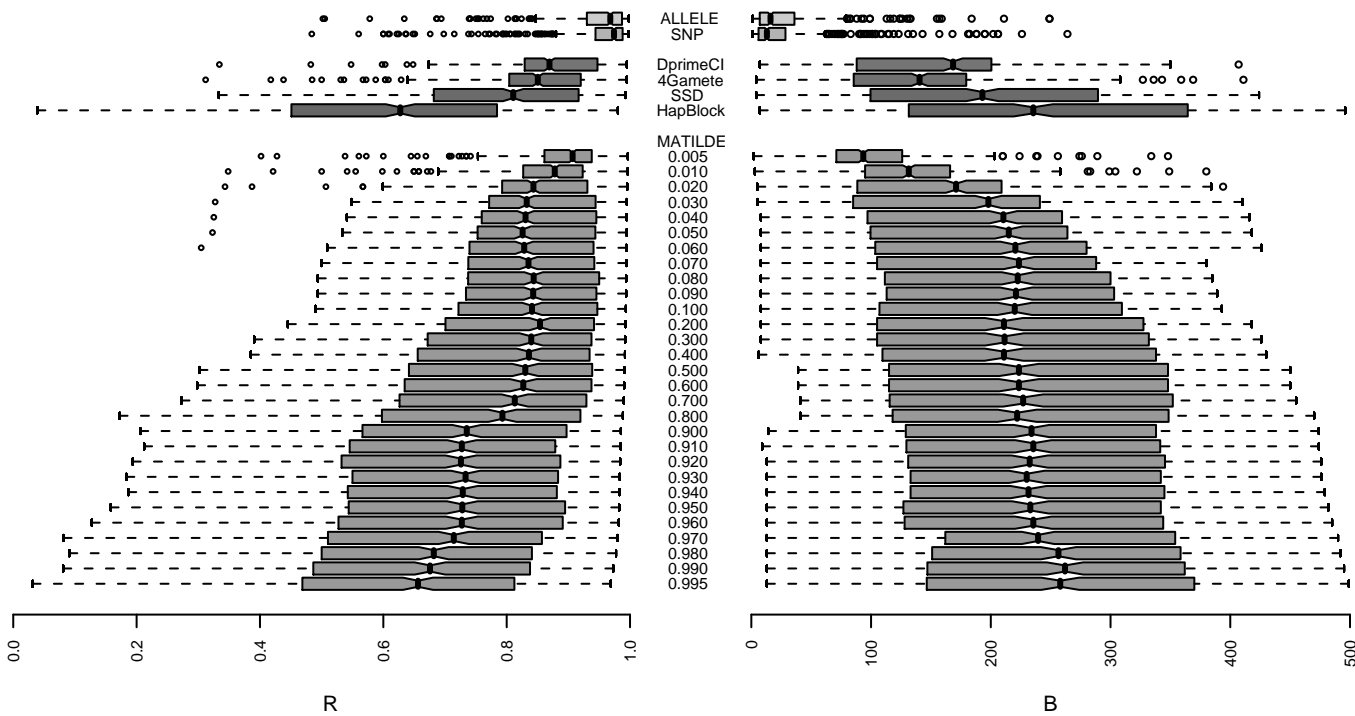

Supplement: Additional file 11 — Parallel distribution of the statistics R (relative position of the block containing the right SNP) and B (number of SNPs belonging to blocks classified not worse than the true SNP) for a sample size of 200 cases and 200 controls. Four effect sizes were considered: the OR is 1.2, 1.4, 1.6 and 1.8, respectively. For each panel, the results of simulation with the allele-based single-SNP method, the genotype-based single-SNP analysis, the four common methods (DprimeCI, 4Gamete, SSD and HapBlock) and the MATILDE at various cutoff thresholds are listed. [file 1471-2164-9-405-S11.pdf]

OR=1.2

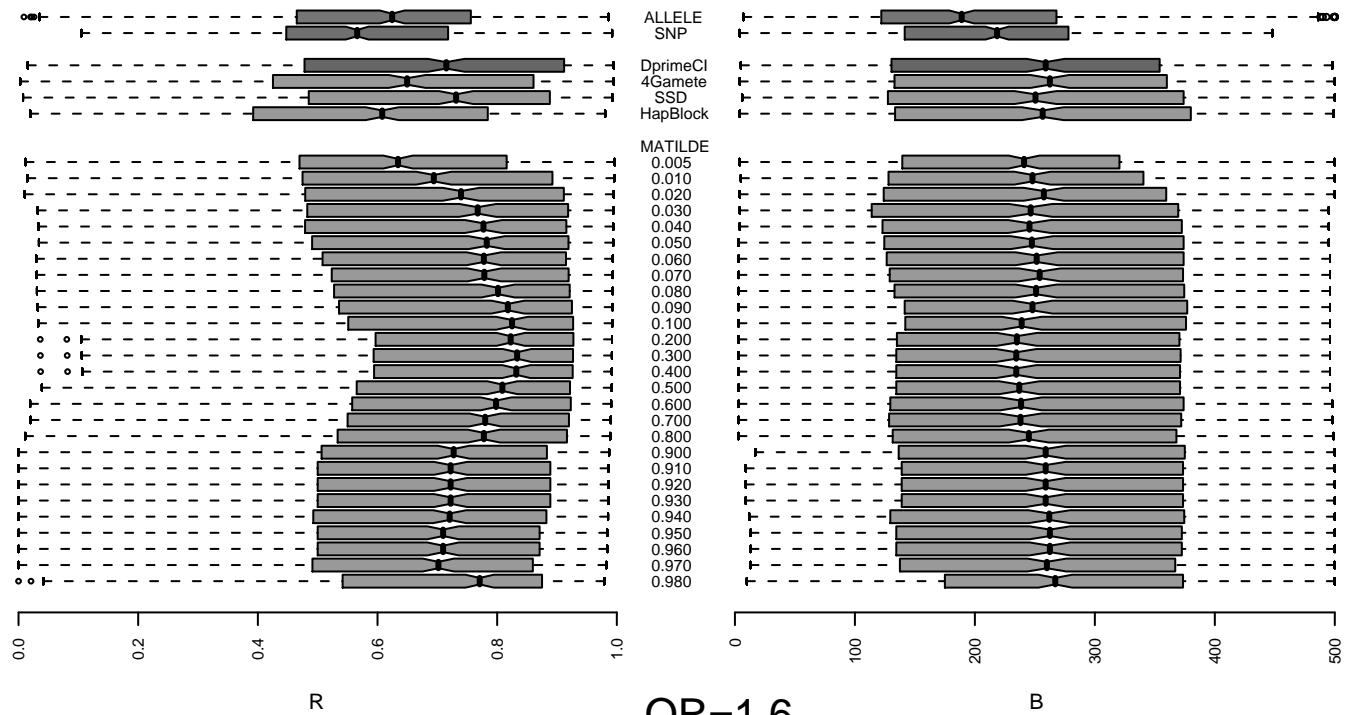

OR=1.4

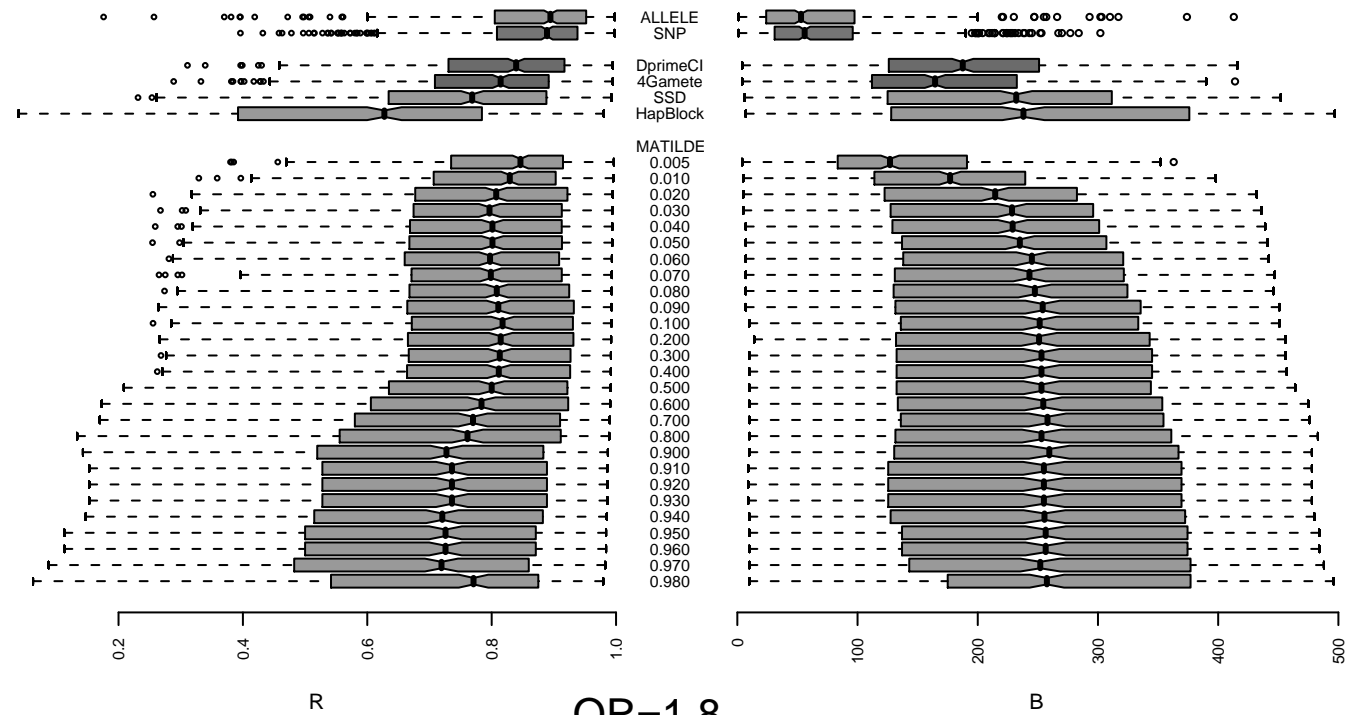

OR=1.6

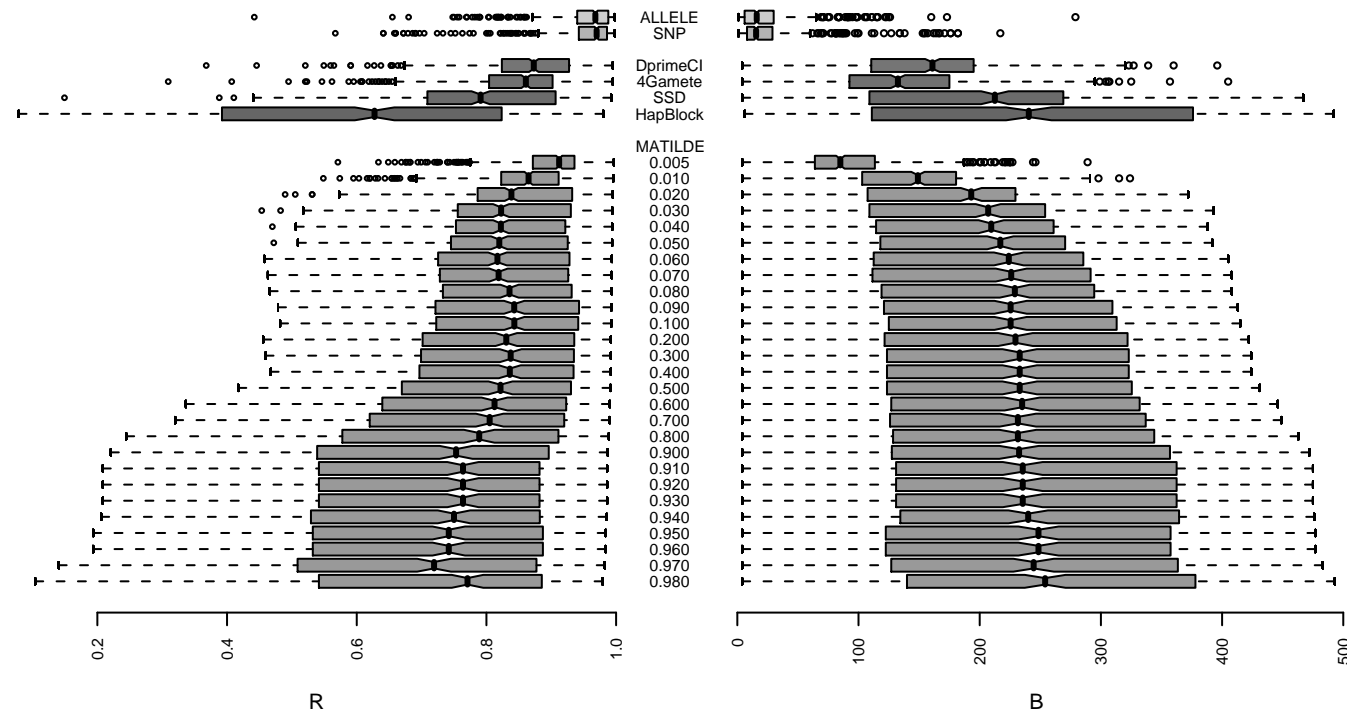

OR=1.8

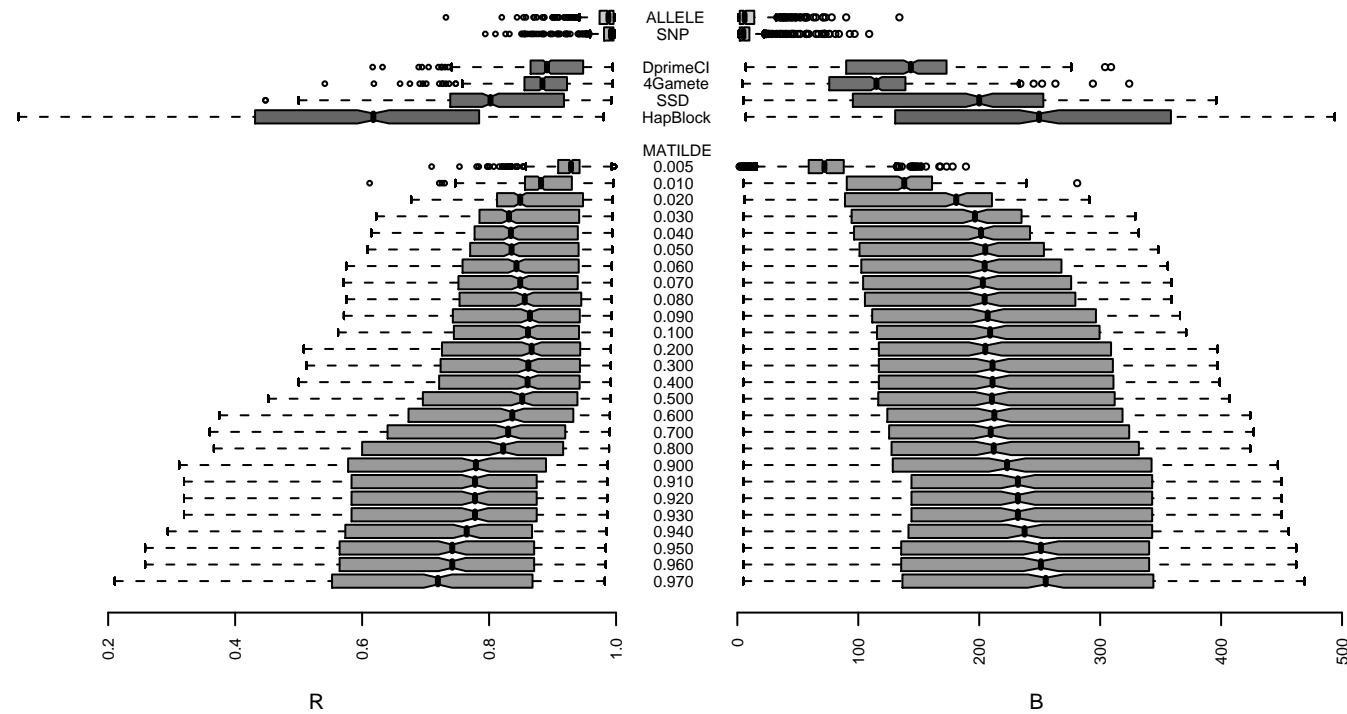

Supplement: Additional file 12 — Parallel distribution of the statistics R (relative position of the block containing the right SNP) and B (number of SNPs belonging to blocks classified not worse than the true SNP) for a sample size of 300 cases and 300 controls. Four effect sizes were considered: the OR is 1.2, 1.4, 1.6 and 1.8, respectively. For each panel, the results of simulation with the allele-based single-SNP method, the genotype-based single-SNP analysis, the four common methods (DprimeCI, 4Gamete, SSD and HapBlock) and the MATILDE at various cutoff thresholds are listed. [file 1471-2164-9-405-S12.pdf]

OR=1.2

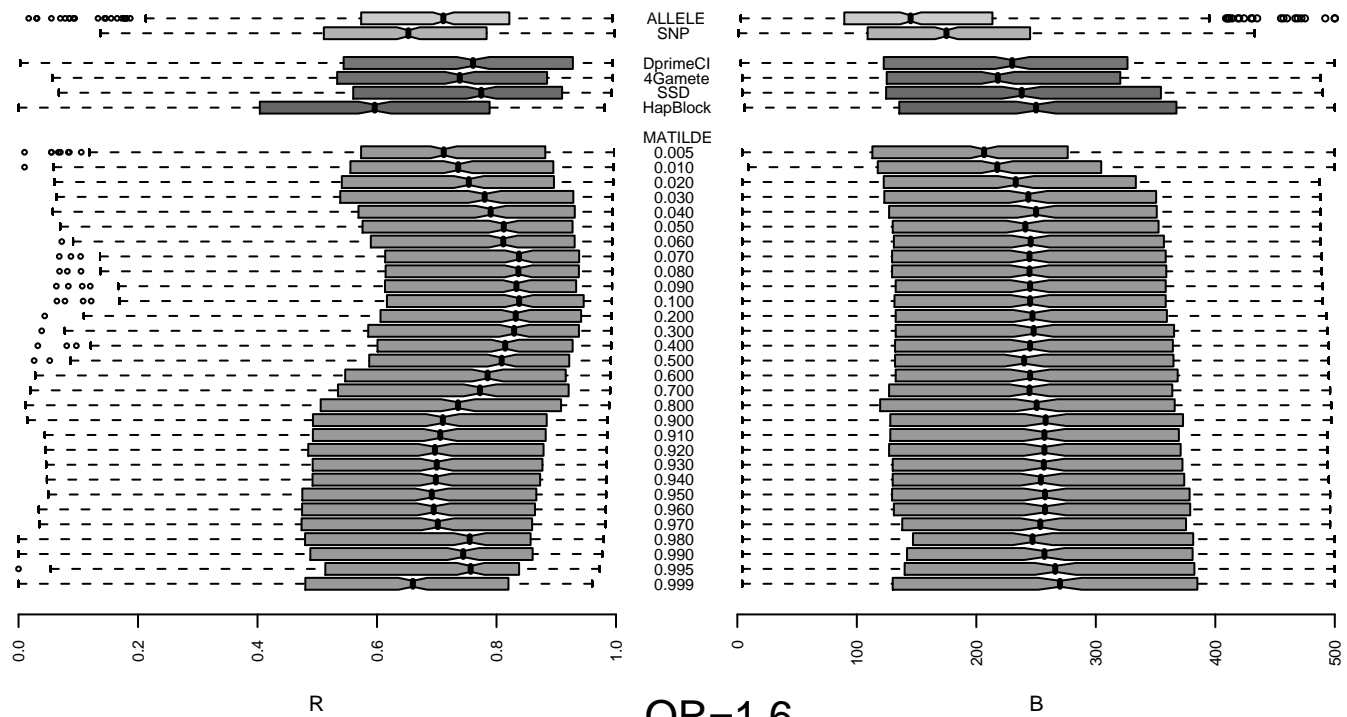

OR=1.4

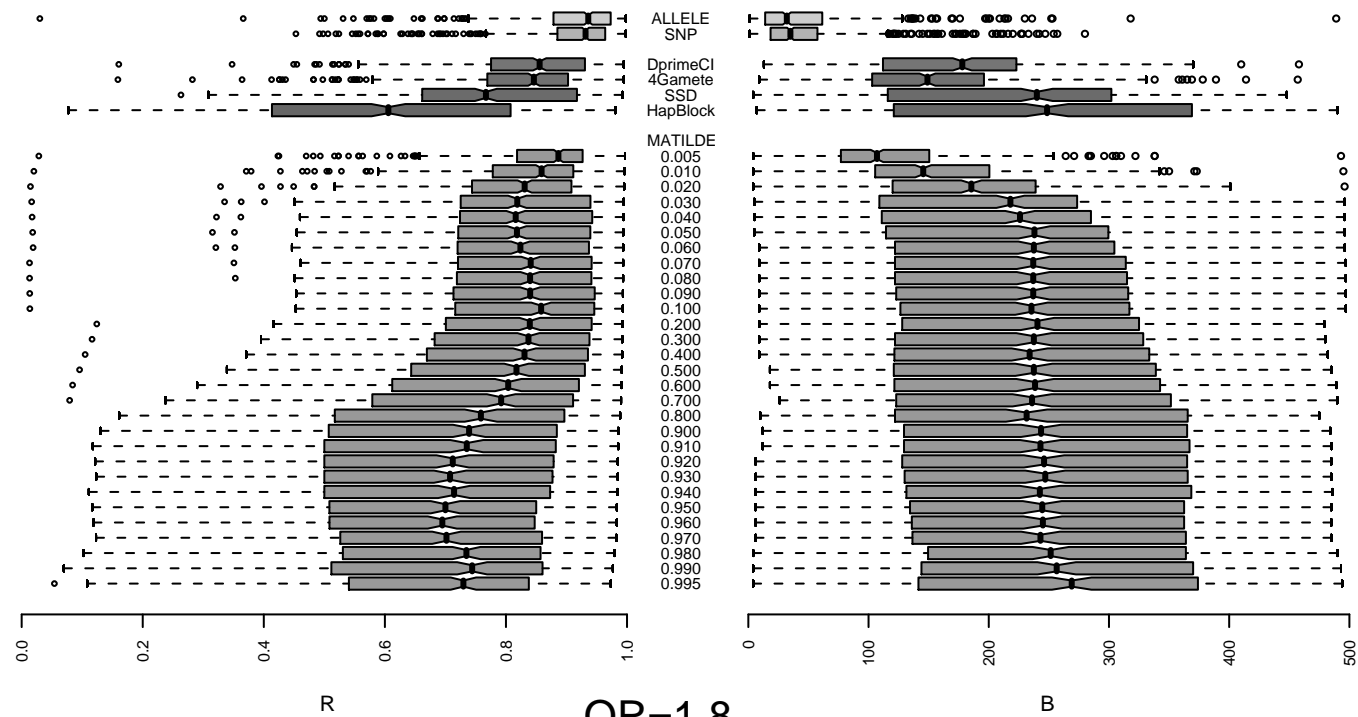

OR=1.6

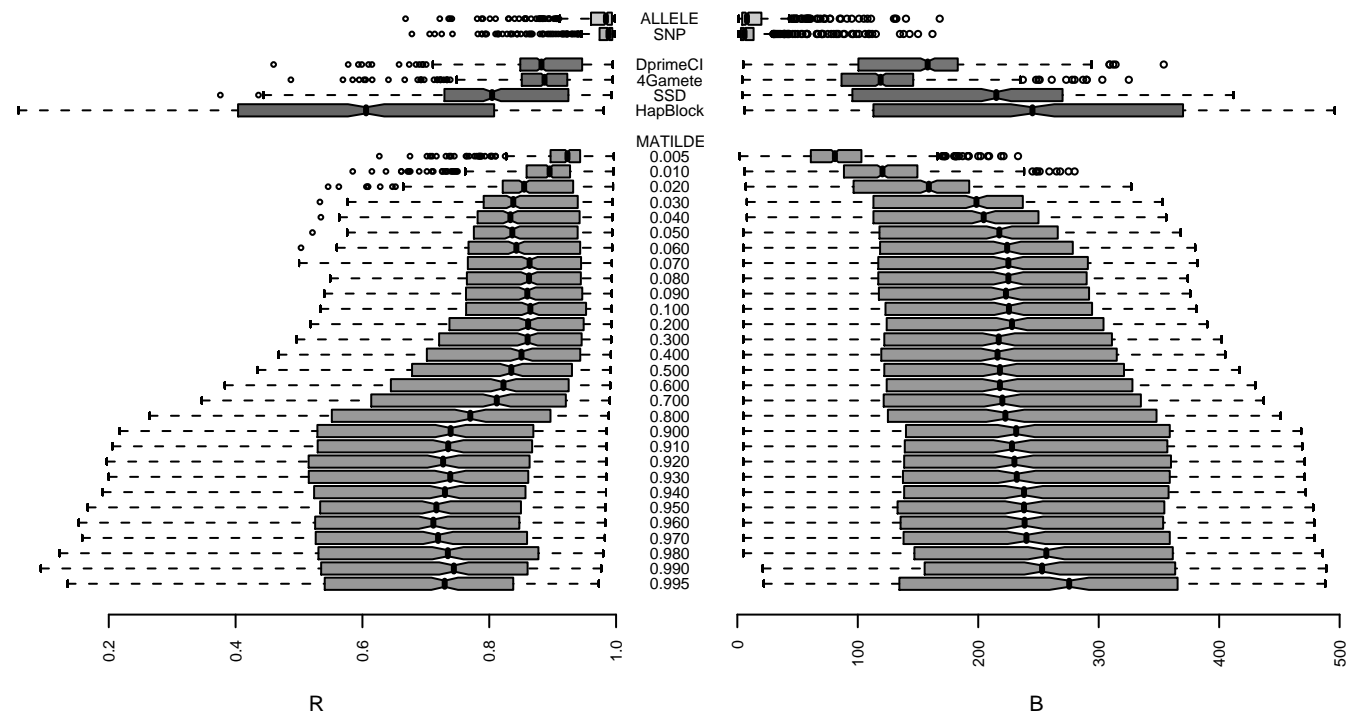

OR=1.8

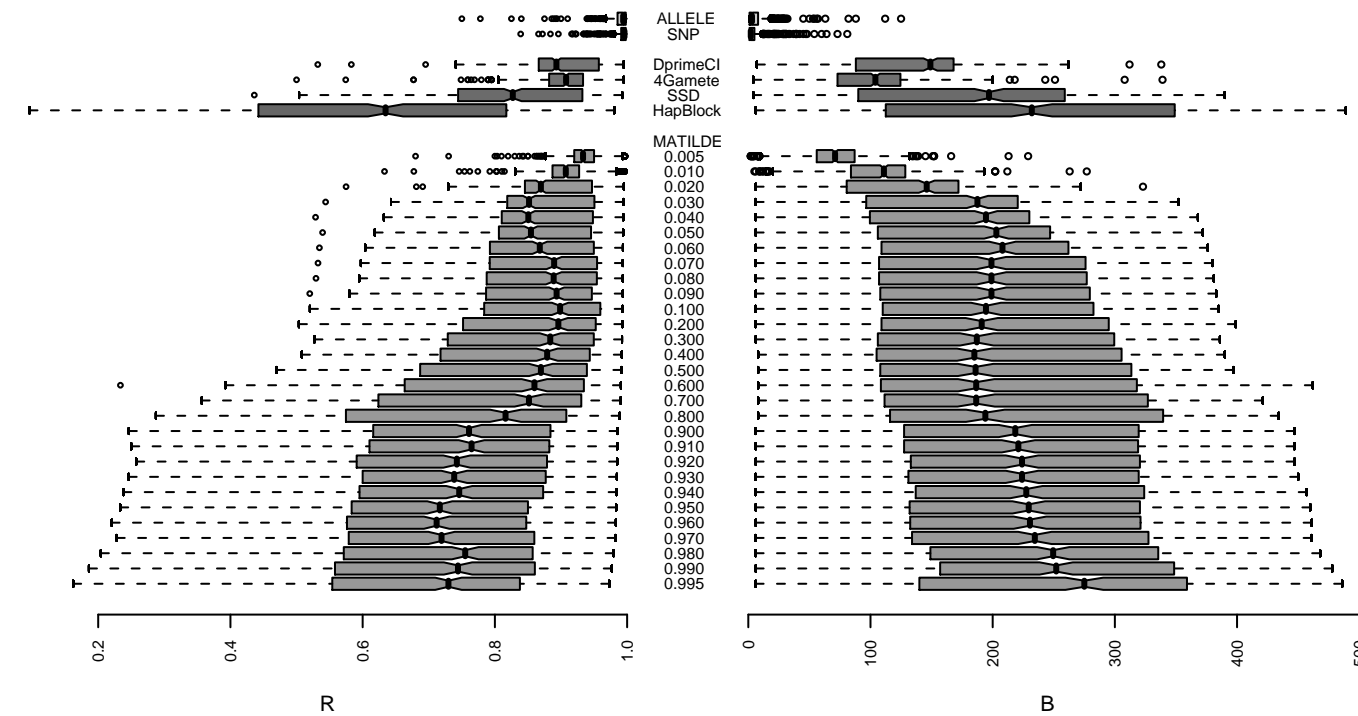

Supplement: Additional file 13 — Parallel distribution of the statistics R (relative position of the block containing the right SNP) and B (number of SNPs belonging to blocks classified not worse than the true SNP) for a sample size of 400 cases and 400 controls. Four effect sizes were considered: the OR is 1.2, 1.4, 1.6 and 1.8, respectively. For each panel, the results of simulation with the allele-based single-SNP method, the genotype-based single-SNP analysis, the four common methods (DprimeCI, 4Gamete, SSD and HapBlock) and the MATILDE at various cutoff thresholds are listed. [file 1471-2164-9-405-S13.pdf]
